# DCAF2 regulates the proliferation and differentiation of mouse progenitor spermatogonia by targeting p21 and thymine DNA glycosylase

**DOI:** 10.1111/cpr.13676

**Published:** 2024-06-04

**Authors:** Hongwei Wei, Zhijuan Wang, Yating Huang, Longwei Gao, Weiyong Wang, Shuang Liu, Yan‐Li Sun, Huiyu Liu, Yashuang Weng, Heng‐Yu Fan, Meijia Zhang

**Affiliations:** ^1^ The Innovation Centre of Ministry of Education for Development and Diseases The second Affiliated Hospital, School of Medicine, South China University of Technology Guangzhou China; ^2^ MOE Key Laboratory for Biosystems Homeostasis and Protection and Innovation Center for Cell Signaling Network Life Sciences Institute, Zhejiang University Hangzhou China

## Abstract

DDB1‐Cullin‐4‐associated factor‐2 (DCAF2, also known as DTL or CDT2), a conserved substrate recognition protein of Cullin‐RING E3 ligase 4 (CRL4), recognizes and degrades several substrate proteins during the S phase to maintain cell cycle progression and genome stability. *Dcaf2* mainly expressed in germ cells of human and mouse. Our study found that *Dcaf2* was expressed in mouse spermatogonia and spermatocyte. The depletion of *Dcaf2* in germ cells by crossing *Dcaf2* ^*fl/fl*^ mice with stimulated by retinoic acid gene 8(*Stra8)*‐Cre mice caused a reduction in progenitor spermatogonia and differentiating spermatogonia, eventually leading to the failure of meiosis initiation and male infertility. Further studies showed that depletion of *Dcaf2* in germ cells caused abnormal accumulation of the substrate proteins, cyclin‐dependent kinase inhibitor 1A (p21) and thymine DNA glycosylase (TDG), decreasing of cell proliferation, increasing of DNA damage and apoptosis. Overexpression of p21 or TDG attenuates proliferation and increases DNA damage and apoptosis in GC‐1 cells, which is exacerbated by co‐overexpression of p21 and TDG. The findings indicate that DCAF2 maintains the proliferation and differentiation of progenitor spermatogonia by targeting the substrate proteins p21 and TDG during the S phase.

## Graphical abstract

Schematic diagram of the CRL4^DCAF2^ ubiquitin complex in mouse progenitor spermatogonia. CRL4^DCAF2^ E3 ligase can promote ubiquitin transfer from RBX‐bound E2 ubiquitin‐conjugating enzymes to the DCAF2 substrate proteins p21 and TDG, promoting their degradation during the S phase. Degradation of these proteins maintains the proliferation and differentiation of progenitor spermatogonia.

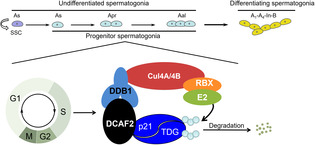

## 1 INTRODUCTION

Spermatogenesis is an extremely complex process. Male germ cells in the testis undergo mitosis, meiosis and sperm deformation to form elongated haploid spermatid.^1^ Spermatogonial stem cells (SSCs) self‐renew to maintain the SSC pool or to generate progenitor spermatogonia for differentiation.^2^ Progenitor spermatogonia undergo differentiation to form differentiating spermatogonia, including type A1, A2, A3, A4, intermediate (In) and B spermatogonia.^3^, ^4^, ^5^ Type B spermatogonia undergo mitosis to form pre‐leptotene spermatocytes, which then enter meiosis to form spermatocytes.^6^ Spermatocytes undergo twice meiosis to form haploid round spermatids.^7^, ^8^ Eventually, round spermatids deform to form elongated spermatids.^7^, ^8^ During the early stages of spermatogenesis, spermatogonia require rapid and precise degradation of cell cycle‐related protein to ensure normal progression of the cell cycle.^9^, ^10^ Disturbed cell cycle progression leads to a larger number of germ cell loss and even Sertoli cell‐only syndrome.^8^, ^11^

Cell cycle progression requires rapid degradation of specific proteins by ubiquitination.^12^ The E3 ubiquitin ligases provide the specificity and selectivity for target protein ubiquitination.^13^, ^14^ CRL4, a key member of the Cullin‐RING E3 ligase (CRL) family, is composed of several essential components, including CUL4A/ B as the central scaffold, damaged DNA‐binding protein 1 (DDB1) serving as a crucial linker and the ring‐finger protein ROC1/2 acting as a recruiter.^15^, ^16^ The CRL4 complex has the ability to interact with over 100 adaptor proteins referred to as DDB1‐CUL4 associated factors (DCAFs) to identify and degrade particular substrate proteins.^17^ For example, DCAF1 targets the cell cycle transcription factor FOXM1 during the S phase.^18^ DCAF3 targets D‐type cyclins^19^ and DCAF8 targets cell division cycle 25A (CDC25A) during the G2/M phase transition.^20^ DCAF11 targets p21 during the G1/S phase transition^21^ and Stem‐loop binding protein (SLBP) during the S phase,^22^ the phosphorylated CENP‐A at Ser68 during the M phase.^23^ DCAF2, also known as DTL or CDT2, targets several substrate proteins to regulate cell cycle progression and maintain genome stability during the S phase, including chromatin licensing and DNA replication factor 1 (CDT1),^24^ checkpoint kinase 1 (CHEK1),^25^ E2F transcription factor 1 (E2F1),^26^ lysine methyltransferase 5A (KMT5A),^27^ p21,^28^, ^29^ SDE2 telomere maintenance homologue 2 (SDE2)^30^ and TDG.^31^ A previous study showed that *Dcaf2* was highest in the mouse testes,^32^ but its function in spermatogenesis remains largely unknown.

In this study, we found that *Dcaf2* expressed in mouse spermatogonia and spermatocyte. Depletion of *Dcaf2* in germ cells through breeding *Dcaf2* ^*fl/fl*^ mice^33^ with *Stra8*‐Cre mice^9^ impaired the cell cycle, proliferation and genome stability in SOX3^+^ progenitor spermatogonia. These functional impairments impede the proliferation of SOX3‐positive (SOX3^+^) progenitor spermatogonia, causing a failure of spermatogonial differentiation (KIT^+^) and meiosis initiation (SYCP3^+^), ultimately resulting in male infertility. Proteomic analysis and western blotting assay showed that depletion of *Dcaf2* led to abnormal accumulation of p21 and TDG. Overexpression of p21 or TDG attenuates proliferation and increases DNA damage and apoptosis in GC‐1 cells, which is exacerbated by co‐overexpression of p21 and TDG. Our data revealed that DCAF2 targets p21 and TDG to maintain the proliferation and differentiation of progenitor spermatogonia.

## 2 MATERIALS AND METHODS

### 2.1 Mice

*Dcaf2* ^*fl/fl*^ mice were generated by the Model Animal Resource Information Platform, Model Animal Research Center of Nanjing University.^33^ Mice carrying the *Dcaf2* ^*fl/fl*^ allele were bred with *Stra8*‐Cre mice.^9^ Genotyping was performed by PCR using mouse tail DNA. The primers used for genotyping are shown in Table S1. The Institutional Animal Care and Use Committee of the South China University of Technology approved all animal protocols (2022102).

### 2.2 RNA extraction and analysis

The RNeasy micro‐RNA isolation kit (Qiagen, Valencia, CA) was used to extract total RNA from the testes, following the manufacturer's instructions. After RNA isolation, reverse transcription was carried out utilizing the QuantiTek reverse transcription system (Qiagen). To determine steady‐state mRNA levels, qRT‐PCR was performed using a Light Cycler 96 instrument (Roche, Basel, Switzerland). The expression of the target genes was evaluated based on the 2^−ΔΔCt^ method, with ribosomal protein L19 (*Rpl19*) serving as the endogenous control. For RNA‐seq analysis, testes were collected from control and cKO mice at postnatal day 9 (P9). RNA was extracted from these samples and GENE DENOVO Technology Co., Ltd. (Guangzhou, China) performed the analysis. The qRT‐PCR primers used are listed in Table S2.

### 2.3 Immunofluorescence and histological analysis

The testes isolated from mice were fixed in 4% paraformaldehyde (PFA) for 24 h at 4°C, embedded in paraffin and sliced into 5‐μm sections. The sections were dewaxed, rehydrated and subjected to antigen retrieval using sodium citrate buffer (0.01 M, pH 6.0). To reduce non‐specific binding, sections were blocked using 10% standard donkey serum. Primary antibodies listed in Table S3 were applied to the sections, followed by treatment with Alexa Fluor 488‐ or 555‐conjugated secondary antibodies (at 1:200 dilution; Thermo Fisher Scientific, MA). DAPI was used for counterstaining and images captured utilizing a Zeiss LSM 800 confocal microscope. For histological analysis, paraffin‐embedded testes were sectioned at a thickness of 5 μm and stained with haematoxylin and eosin (H&E).

### 2.4 Western blotting and proteomic analysis

The testes' total proteins were extracted using RIPA buffer (Beyotime, Shanghai, China), and 20 μg protein was used for sample loading. SDS‐PAGE was used to separate the protein samples, which were then transferred to a PVDF membrane (Millipore, Darmstadt, Germany). The membranes were obstructed in a mixture of TBST (Tris‐buffered saline with Tween 20) buffer containing 5% milk and were subsequently placed in contact with primary antibodies (Table S3) overnight at 4°C. Following a wash with TBST buffer, the membranes were subjected to secondary antibodies at an appropriate dilution (1:10,000; Zhongshan Golden Bridge Biotechnology, Beijing, China). Eventually, the bands were identified utilizing the SuperSignal West Pico Kit (Thermo Fisher Scientific) and displayed through a Tanon 5200 chemiluminescent imaging system (Tanon, Shanghai, China). The relative intensities of the bands were calculated using the ImageJ software (NIH Image, Bethesda, MD). GAPDH was utilized as an internal reference. Unaltered scans of exemplary blots can be found in the supplementary original blots. For proteomic analysis, testes were collected from cKO and control mice at P9. Proteins were extracted from the samples and analysed by GENE DENOVO Technology Co., Ltd.

### 2.5 TUNEL assay

A Click‐iT Plus TUNEL Assay (1982275; Thermo Fisher Scientific) was utilized to identify apoptotic cells in the study. Initially, the rehydrated sections underwent fixation with a 4% PFA solution. Following this step, permeabilization was achieved with 0.5% Triton X‐100, and the sections were then subjected to the TUNEL reaction mixture. Subsequent to PBS washing, DAPI was used for counterstaining. The Zeiss LSM 800 confocal microscope was employed to visualize the incorporated fluorescence.

### 2.6 Leydig cells isolation

Leydig cells were acquired from P21 mice using a modified approach based on a previously published technique.^34^ In short, the testes were digested with collagenase II (C6885; Sigma‐Aldrich, Germany). The resultant cells were filtered and then layered on top of gradients of 60%, 37%, 26% and 21% Percoll (P4937; Sigma‐Aldrich). After centrifugation at 3000*g* for 30 min at 4°C, Leydig cells were harvested from the layer between 60% and 37% Percoll. Verification of the purity of the isolated Leydig cells was carried out through HSD3β immunofluorescence staining.

### 2.7 5‐Bromo‐2‐deoxyuridine incorporation

For 5‐bromo‐2‐deoxyuridine (BrdU) incorporation experiments, mice received intraperitoneal injections of 10 μL/g body weight of 10 mg/mL BrdU (B5002; Sigma‐Aldrich) in PBS, 4 h before they were sacrificed.^35^

### 2.8 In vivo BTB integrity analysis

The BTB integrity was assessed following a previously described protocol.^36^ To summarize, under anaesthesia, mice's unilateral testes were exposed and a thin needle was introduced into the testicular interstitium, followed by administering 20 μL of Biotin (10 mg/mL) dissolved in PBS with 1 mM CaCl_2_ (F3272; Sigma‐Aldrich). After 30 min, the testes were excised, embedded in optimal cutting temperature compound (4583; Tissue‐Tek, Maryland), and sliced into 10‐μm sections using a cryostat. Subsequently, these sections were rinsed with PBS and incubated in a solution of FITC‐labelled streptavidin (Zhong Shan Jin Qiao) at a 1:200 dilution for 1 h at 37°C. Nuclei were counterstained with DAPI.

### 2.9 Cell culture, plasmids and transfection

A mouse spermatogonia cell line (GC‐1 cells) was purchased from the Procell Life Science & Technology (Wuhan, China) and cultured in a humidified incubator at 37°C in 5% CO_2_. The cells were cultured in Dulbecco's modified Eagle's medium containing 1% penicillin–streptomycin solution (NCM Biotech, China) and 10% foetal bovine serum (10091148; Thermo Fisher Scientific).

According to the manufacturer's instructions, the X‐treme GENE HP DNA Transfection Reagent (Roche) was used to transfected the following plasmids: pcDNA3.1‐NC (negative control; empty vector), pcDNA3.1‐EGFP‐p21 (plasmid expressing EGFP‐labelled p21) and pSC2‐mCherry‐TDG (plasmid expressing mCherry‐labelled TDG), which were constructed and purchased from Beijing Genomics Institute (Beijing, China).

### 2.10 Cell proliferation assays

For the cell counting kit 8 (CCK‐8) assays, equal amounts of GC‐1 cells were added to 96‐well plates. After 48 h of transfection, cell viability was evaluated using CCK‐8 (C0041; Beyotime, Shanghai, China) and a microplate reader (Bio‐Rad) at an optical density of 450 nm, as previously reported.^37^

### 2.11 Immunoprecipitation

Mouse testes at P9 were dissected and extracted in RIPA buffer, clarified by centrifugation at 12,000*g*. Then, according to the manufacturer's instructions, immunoprecipitation was performed using Dynabeads™ Protein A immunoprecipitation kit (10006D; Thermo Fisher Scientific). In general, antibodies bind to beads. Protein extracts were incubated with antibody‐bound beads at 4°C with rotation. The beads were washed with washing buffer and then denaturing elution was performed. The eluted protein samples were subjected to Western blotting, and the corresponding antibodies were incubated. For ubiquitination and Co‐IP assays, protein samples were preincubated with beads conjugated normal rabbit/mouse IgG.

### 2.12 Statistical analysis

To ensure accuracy, each experiment was repeated a minimum of three times. Subsequently, the obtained data underwent analysis and was visualized with the aid of GraphPad Prism software (v8.3.0, La Jolla, CA). The data represent the mean ± SEM. The *p*‐values was determined by two‐tailed unpaired Student's *t*‐tests unless otherwise stated (*p < 0.05, **p < 0.01, ***p < 0.001, ns; no significance).

## 3 RESULTS

### 3.1 *Dcaf2* depletion in germ cells causes male sterility

**FIGURE 1:**
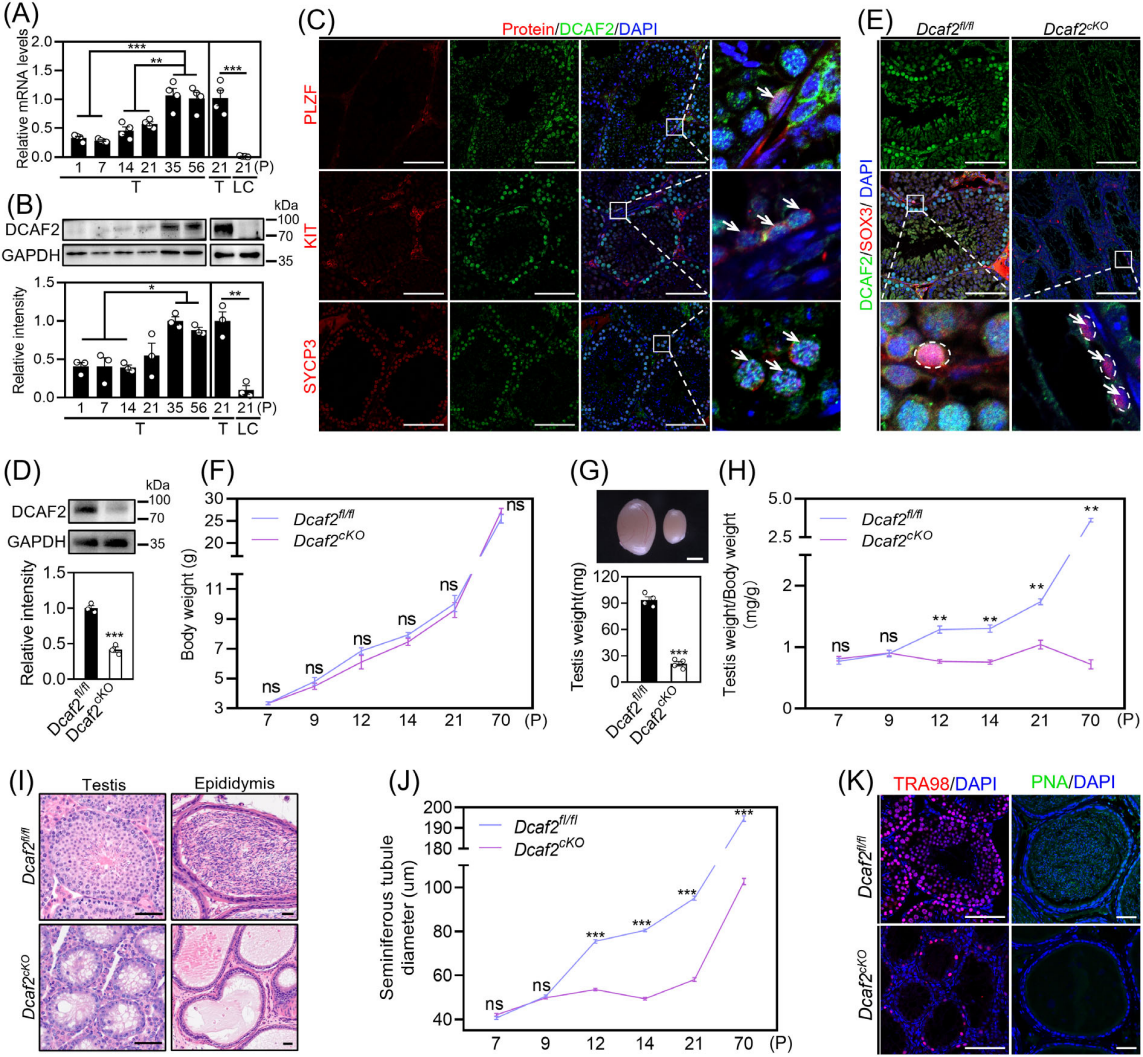
*Dcaf2* depletion in germ cells causes male sterility. (A, B) The mRNA (A) and protein (B) levels of DCAF2 in mouse different stages. GAPDH was used as an internal control. (C) Double‐immunofluorescence staining of seminiferous tubule sections from adult mouse testes. DCAF2 (green), PLZF (an undifferentiated spermatogonia marker, red), KIT (a differentiating spermatogonia marker, red) and SYCP3 (a spermatocyte marker, red). White arrowheads indicate representative double‐positive spermatogonia or spermatocytes. Nuclei were counterstained with DAPI (blue). (D) Detection of *Dcaf2* knockout efficiency in the adult testes using western blotting. (E) Detection of *Dcaf2* knockout efficiency in the adult testes using double‐immunofluorescence staining of DCAF2 (green) and SOX3 (red). Dotted circles indicated representative SOX3^+^ progenitor spermatogonia; White arrowheads indicate representative DCAF2^−^SOX3^+^ progenitor spermatogonia. (F–H) The compare of body weight (F), and testis morphology at P70 (G) and the testicular index (H) in cKO and control mice. (I) Haematoxylin and eosin (H&E) staining of testis and cauda epididymis in adult cKO and control mice. (J) Seminiferous tubule diameter curve. (K) Immunofluorescent staining of TRA98 (a germ cell marker, red) in the testis and PNA (an acrosome of spermatid marker, green) in the cauda epididymis of adult mice. T, testes; LC, Leydig cells. For A, B, E F, G and H, *n* = 3–4 independent experiments. For J, more than 200 tubules from 4 independent experiments were scored in each group. Scale bars, C, D, E and K, 100 μm; G, 2000 μm; I, 50 μm. The *p‐*values were determined by one‐way ANOVA followed by Tukey's test (A, B). *p < 0.05, **p < 0.01, ***p < 0.001, ns; no significance.

A previous study showed that *Dcaf2* was highest in the mouse testes.^32^ We further investigated the spatiotemporal expression patterns of DCAF2 in the mouse testes. The findings indicated a gradual increase in both the mRNA and protein levels of DCAF2 from P1 to P35 (Figure 1A,B). Moreover, the mRNA and protein levels of DCAF2 in the mouse testes were significantly higher than those in Leydig cells (Figure 1A,B), consistent with the results in human testis showing that *Dcaf2* mRNA is mainly expressed in germ cells (Figure S1A,B). Next, we determined the localisation of DCAF2 during spermatogenesis by co‐staining DCAF2 with promyelocytic leukaemia zinc finger (PLZF), kit proto‐oncogene receptor tyrosine kinase (KIT), or synaptonemal complex protein 3 (SYCP3) in adult testes. The results showed that DCAF2 was mainly localized in the nucleus of undifferentiated (PLZF^+^ cells) and differentiating (KIT^+^ cells) spermatogonia, as well as in the nucleus of meiotic pro‐phase I spermatocyte (SYCP3^+^ cells) (Figure 1C).

To determine the function of DCAF2 during spermatogenesis, we generated *Dcaf2*‐conditional knockout (hereafter referred to as cKO) mice by crossing *Dcaf2* ^*fl/fl*^ mice (hereafter referred to as control) with *Stra8*‐Cre mice.^9^ The depletion of *Dcaf2* in mouse testes was confirmed using western blotting (Figure 1D). The immunofluorescence co‐staining of DCAF2 with SOX3 and GFRα1 in the mouse testes suggested that *Dcaf2* was deleted in progenitor spermatogonia (SOX3^+^), but not in spermatogonial stem cells (GFRα1^+^) (Figures 1E and S2E). The cKO males exhibited normal physical appearance and survival but were found to be sterile (Figure 1F). This infertility was reflected in a significant decrease in testis weight in cKO mice compared with the controls, starting from P12 (Figures 1G,H and S2B). Histological analysis of adult cKO mice revealed abnormal seminiferous tubules characterized by atrophy and a lack of germ cells, with an abundance of vacuoles present (Figures 1I,J and S2C,D). Immunostaining showed a depletion of many germ cells, with no haploid spermatids observed in the cKO testes (Figure 1K). This indicated a severe disruption in spermatogenesis in the cKO mice, leading to complete sterility. Taken together, these results indicated that *Dcaf2* is indispensable for spermatogenesis.

### 3.2 *Dcaf2* depletion decreases the number of progenitor spermatogonia

**FIGURE 2:**
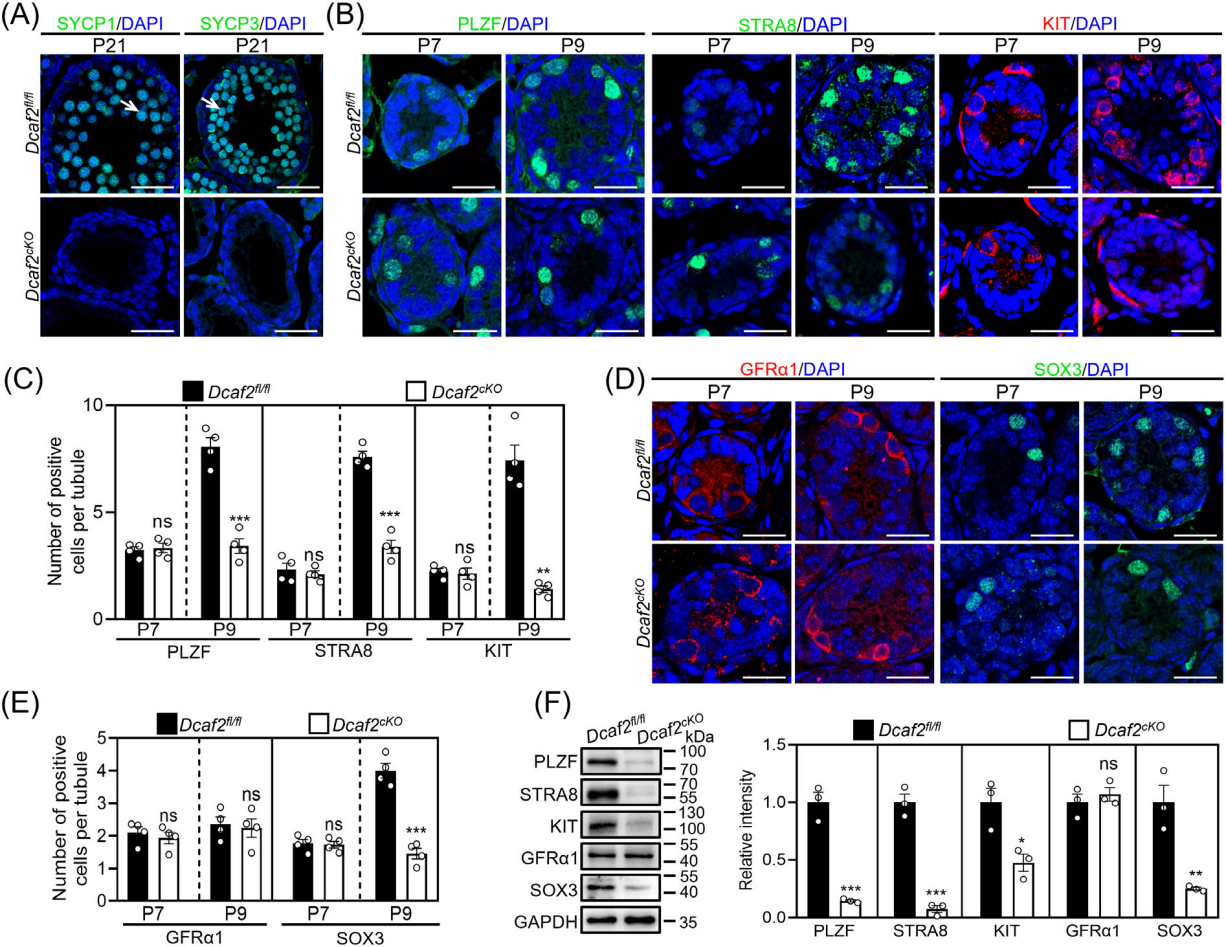
SOX3^+^ progenitor spermatogonia are decreased in cKO testes. (A) Immunofluorescent staining of SYCP3 and SYCP1 in cKO and control testes at P21. White arrowheads indicate representative SYCP3^+^ or SYCP1^+^ spermatocytes. (B) Immunofluorescent staining of PLZF, STRA8 and KIT in cKO and control testes at P7 and P9. (C) Numbers of PLZF^+^, STRA8^+^ and KIT^+^ cells per seminiferous tubule in cKO and control testes at P7 and P9. (D) Immunofluorescent staining of GFRα1 and SOX3 in cKO and control testes at P7 and P9. (E) Numbers of GFRα1^+^ and SOX3^+^ cells per seminiferous tubule in cKO and control testes at P7 and P9. (F) Western blot analysis of PLZF, STRA8, KIT, GFRα1 and SOX3 levels in cKO and control testes at P9. GAPDH was used as an internal control. Nuclei were counterstained with DAPI (blue). Scale bars; 25 μm. For F, *n* = 3 independent experiments. For C and E, more than 200 tubules from four independent experiments were scored in each group. *p < 0.05, **p < 0.01, ***p < 0.001, ns; no significance.

To determine whether spermatogonia could enter into meiotic spermatocytes in cKO testes, we performed immunofluorescence staining for the meiosis markers SYCP1 and SYCP3 in P14 and P21 testis sections. The results showed that no spermatocytes were detected in the cKO testes (Figures 2A and S3A,B), indicating that *Dcaf2* depletion caused a severe defect in meiotic initiation during spermatogenesis. The meiotic initiation requires several rounds of mitotic division in spermatogonia.^6^, ^38^ Thus, we examined the number of spermatogonia by staining for different spermatogonia markers in P7 and P9 testis sections. The results showed that the number of undifferentiated (PLZF^+^ cells) and differentiating (STRA8^+^ or KIT^+^ cells) spermatogonia was significantly decreased in cKO testes compared with the controls (Figures 2B,C and S4A,B). The undifferentiated spermatogonia are subdivided into SSCs and progenitor spermatogonia.^9^, ^39^ In our study, the number of progenitor spermatogonia (SOX3^+^ cells), but not SSCs (GFRα1^+^ cells), was significantly decreased in cKO testes compared with the controls (Figures 2D,E and S5A–D). Consistent with this, the protein levels of PLZF, STRA8, KIT and SOX3, but not GFRα1, were significantly decreased in cKO testes compared with the controls (Figure 2F). However, SSCs accumulated in the basement membrane of seminiferous tubules from the cKO adult testes compared with the controls (Figure S6A,B). The findings indicate that *Dcaf2* depletion result in a reduction of progenitor spermatogonia, which could further result in a reduction of differentiating spermatogonia.

We examined the function of Sertoli cells in cKO testes. The results showed that the number of Sertoli cells (SOX9^+^ cells) and the protein levels of SOX9 and E‐cadherin did not differ between cKO and control testes (Figure S7A,B). Furthermore, immunofluorescence staining of blood‐testes barrier (BTB)‐related proteins (vimentin, tight junction protein 1 [TJP1, also known as ZO‐1], claudin‐11 and connexin 43) and a biotin tracing assay showed that the function of BTB was complete in the cKO testes (Figure S7C).

### 3.3 *Dcaf2* depletion results in abnormal accumulation of p21 and TDG

**FIGURE 3:**
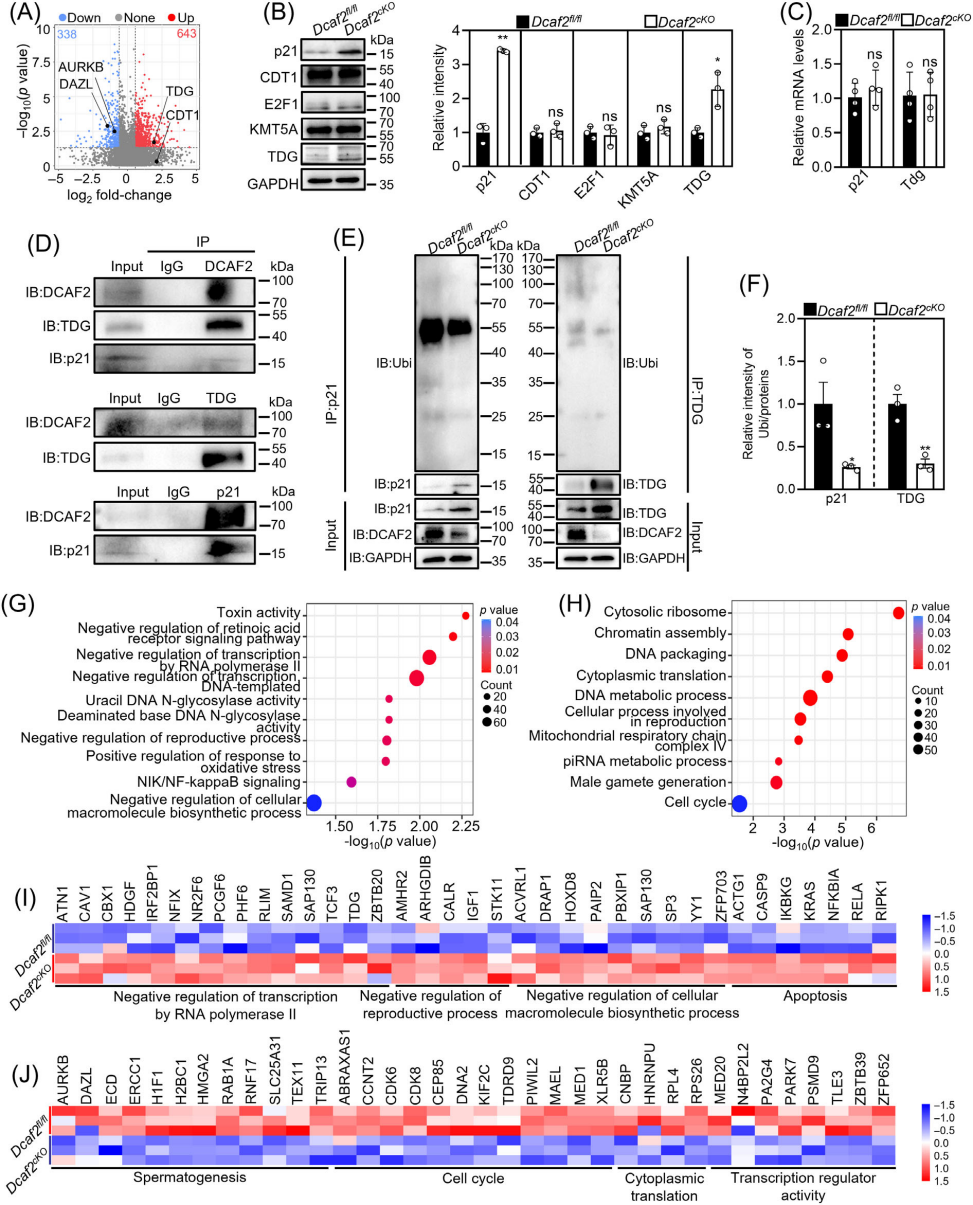
*Dcaf2* depletion in male germ cells increases the protein levels of p21 and TDG. (A) Volcano plot illustrating differentially expressed proteins in cKO and control testes. (B) Western blot analysis of p21, CDT1, E2F1, KMT5A and TDG levels in cKO and control testes at P9. GAPDH was used as an internal control. (C) mRNA levels of *p21* and *Tdg* in cKO and control testes at P9. (D) Western blot analysis showed that there was an interaction in DCAF2 with p21 and TDG protein in mouse testis tissue. (E, F) ubiquitination assay showed that depletion of *Dcaf2* decreased the ubiquitination levels of p21 and TDG. (G, H) Bubble chart illustrating the enriched GO terms associated with significantly upregulated (G) and downregulated (H) proteins in cKO and control testes identified using DIA proteomics. Proteins with a fold‐change ≥1.5 and a *p‐*value <0.05 were selected for analysis. (I, J) Heatmaps illustrating the differences in the expression of upregulated (I) and downregulated (J) proteins involved in various processes between cKO and control testes. For B, C and F, *n* = 3–4 independent experiments. *p < 0.05, **p < 0.01, ns; no significance.

Previous studies have demonstrated that DCAF2 targets several substrate proteins.^40^ Proteomic analysis showed that 643 proteins (including TDG) were significantly upregulated, 338 proteins were significantly downregulated and 8503 proteins (including CDT1, CHEK1, FBH1 and SDE2) were not significantly different in cKO testes compared with the controls (Figures 3A and S8A). Other DCAF2 substrate proteins, including E2F1, KMT5A and p21, were not detected in the proteome. These changes in the expression of representative proteins were validated using western blotting (Figures 3B and S8B). Western blotting and qRT‐PCR assays showed that the protein, but not the mRNA, levels of p21 and TDG were significantly increased in cKO testes compared with the controls (Figure 3B,C). DCAF2 interacts with p21 and TDG to promote their ubiquitination and degradation in Hela cells.^28^, ^31^ Thus, we performed the Co‐IP and ubiquitination assay using P9 mouse testes, in which almost all germ cells are spermatogonia. The Co‐IP assay verified the interaction in DCAF2 with TDG and p21 (Figure 3D), and the ubiquitination assay showed that the depletion of *Dcaf2* decreased the ubiquitination levels of p21 and TDG (Figure 3E,F). These results indicate that DCAF2 can interact with p21 and TDG to promote their ubiquitination and degradation in mouse spermatogonia. Protein enrichment analysis showed that the upregulated proteins in the cKO testes were mainly related to the negative regulation of RNA polymerase II‐dependent transcription, reproduction, cellular macromolecule biosynthesis and apoptosis (Figure 3G,H). The downregulated proteins in the cKO testes mainly controlled spermatogenesis, cell cycle, cytoplasmic translation and transcription regulator activity (Figure 3I,J). These functional impairments may contribute to defects in proliferation and differentiation of progenitor spermatogonia.

### 3.4 *Dcaf2* depletion disturbs the expression of genes involved in progenitor spermatogonia

**FIGURE 4:**
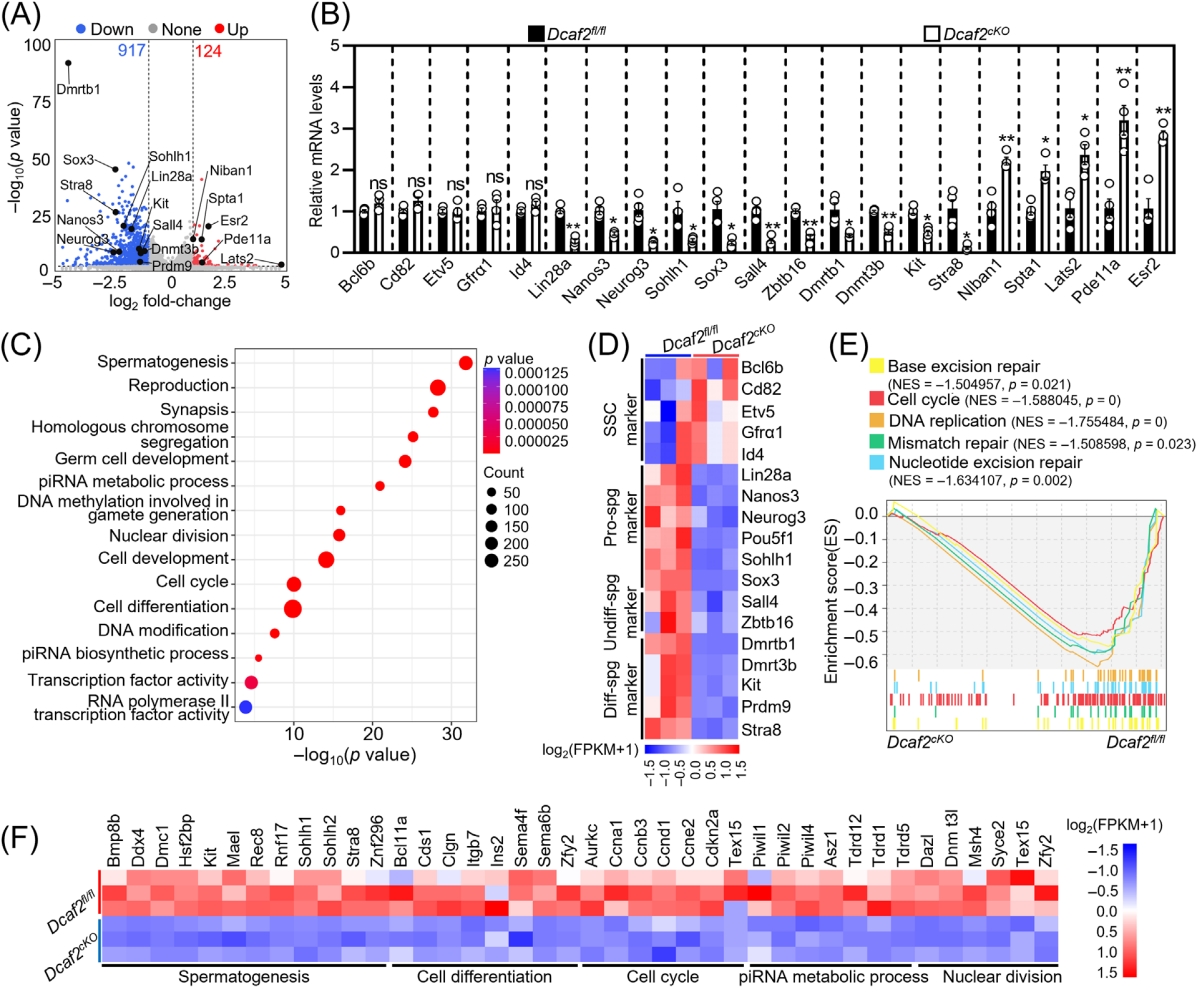
*Dcaf2* depletion disturbs the expression of genes involved in progenitor spermatogonia. (A) Volcano plot illustrating differentially expressed transcripts in cKO and control testes. (B) Quantitative RT‐PCR validating changes in representative transcripts selected from the RNA‐seq data. *n* = 4 independent experiments. (C) Bubble chart illustrating the enriched GO terms associated with the significantly downregulated transcripts identified by RNA‐seq in cKO and control testes. Transcripts with a fold‐change ≥2 and a *p‐*value 0.05 were selected for analysis. (D) Heatmap showing the mRNA abundance of genes functioning in spermatogonial stem cells (SSCs; e.g., *Bcl6b*, *Etv5*, *Cd82*, *Id4* and *Gfrα1*), progenitor spermatogonia (pro‐spg; e.g., *Lin28a*, *Nanos3*, *Neurog3*, *Pou5f1*, *Sohlh1* and *Sox3*), undifferentiated spermatogonia (Undiff‐spg; e.g., *Sall4* and *Zbtb16*), and differentiating spermatogonia (Diff‐spg; e.g., *Dmrtb1*, *Dnmt3b*, *Kit*, *Stra8* and *Prdm9*). (E) Gene set enrichment analysis (GSEA) revealing enrichment of the cell cycle, DNA replication and repair in cKO testes relative to the controls. NES, normalized enrichment score. (F) Heatmap illustrating the differences in the expression of downregulated transcripts involved in various processes between cKO and control testes. *p < 0.05, **p < 0.01, ***p < 0.001, ns; no significance.

To further determine the mechanism of action of DCAF2 in spermatogenesis, we performed transcriptomic analysis of cKO and control testes. In cKO testes, there were a total of 1041 transcripts showing differential expression compared with the controls, with 124 upregulated and 917 downregulated transcripts (Figure 4A). Validation of these changes in transcript expression was carried out through qRT‐PCR for representative transcripts (Figure 4B). Gene enrichment analysis revealed that the downregulated transcripts in the cKO testes mainly controlled spermatogenesis, cell cycle, cell differentiation, nuclear division, DNA replication and repair (Figures 4C,E and S9A,B). Analysis of several spermatogonia‐associated marker genes showed that genes associated with progenitor spermatogonia, undifferentiated spermatogonia and differentiating spermatogonia were significantly downregulated in the cKO testes compared with the controls. However, marker genes associated with SSCs maintenance showed minimal changes in the cKO testes (Figure 4D). These findings indicate that *Dcaf2* depletion interfered with genes crucial for progenitor spermatogonia, influencing their proliferation and differentiation.

### 3.5 Correlation between protein expression and mRNA expression in cKO testes

**FIGURE 5:**
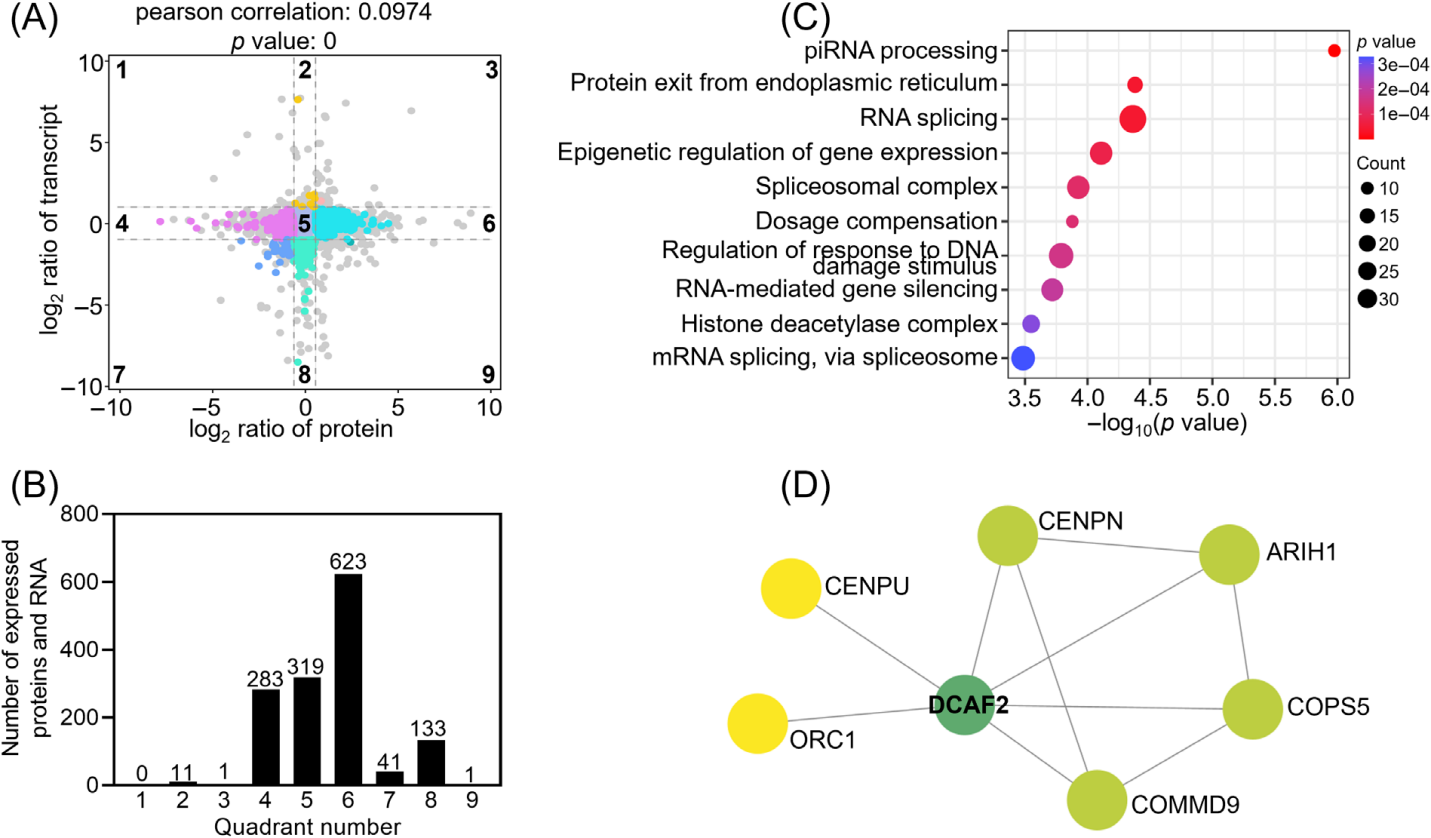
Correlation between protein and mRNA expression in cKO testes relative to the controls. (A) Correlated proteins and RNAs were enriched in nine quadrants of the cKO testes. Quadrants 1, 2 and 4 indicate that protein abundance was lower that of RNA. In quadrants 3 and 7, the RNAs correspond with the related proteins. Quadrant 5 showed that proteins and RNAs were commonly expressed with no differences. Quadrants 6, 8 and 9 indicate that the protein abundance was higher than the RNA abundance (if the fold change was reached and the *p*‐value was not reached, it is shown as a grey point). (B) Number of RNAs and proteins enriched in the nine quadrants. (C) Bubble chart illustrating enriched GO terms associated with genes in quadrants 6, 8 and 9. (D) Protein–protein interaction network (PPI) analysis was performed for genes in quadrants 6, 8 and 9.

We performed a nine‐quadrant analysis using the Omicshare platform (http://www.omicshare.com) to examine the relationship between proteins and RNAs in the cKO testes. A lower Pearson correlation indicated that there was little correlation between proteome and transcriptome. Genes were highly enriched in quadrant 6, followed by in quadrants 5 and 4. The proteins in quadrants 6, 8 and 9 showed higher abundances than the related RNAs, which might be due to post‐transcriptional or translation‐level regulation. A small number of proteins in quadrants 1, 2 and 4 showed a lower abundance than the related RNAs. The enrichment of proteins and RNAs in quadrants 3 and 7 showed similar expression patterns. The enrichment of proteins and RNAs in quadrant 5 indicated that these proteins and RNAs were commonly expressed with no differences (Figure 5A,B). The functional enrichment analysis revealed that the genes in quadrants 6, 8 and 9 were primarily involved in piRNA processing, the spliceosomal complex, regulation of the response to DNA damage stimulus and dosage compensation (Figure 5C). These functional impairments may also contribute to defects in proliferation and differentiation of progenitor spermatogonia.

To identify potential DCAF2 substrate proteins, we integrated 757 genes in quadrants 6, 8 and 9 into protein–protein interaction networks using the STRING database (https://cn.string-db.org/). This result shows that ARIH1, CENPU, COSP5, COMMD9, ORC1 and CENPN were predicted to interact with DCAF2 (Figure 5D). ORC1 was also predicted to interact with proliferating cell nuclear antigen (PCNA), which is involved in DCAF2 targeting substrate proteins.^41^ Thus, these proteins, particularly ORC1, may be potential substrates of DCAF2.

### 3.6 *Dcaf2* depletion impairs proliferation and genome stability of progenitor spermatogonia

**FIGURE 6:**
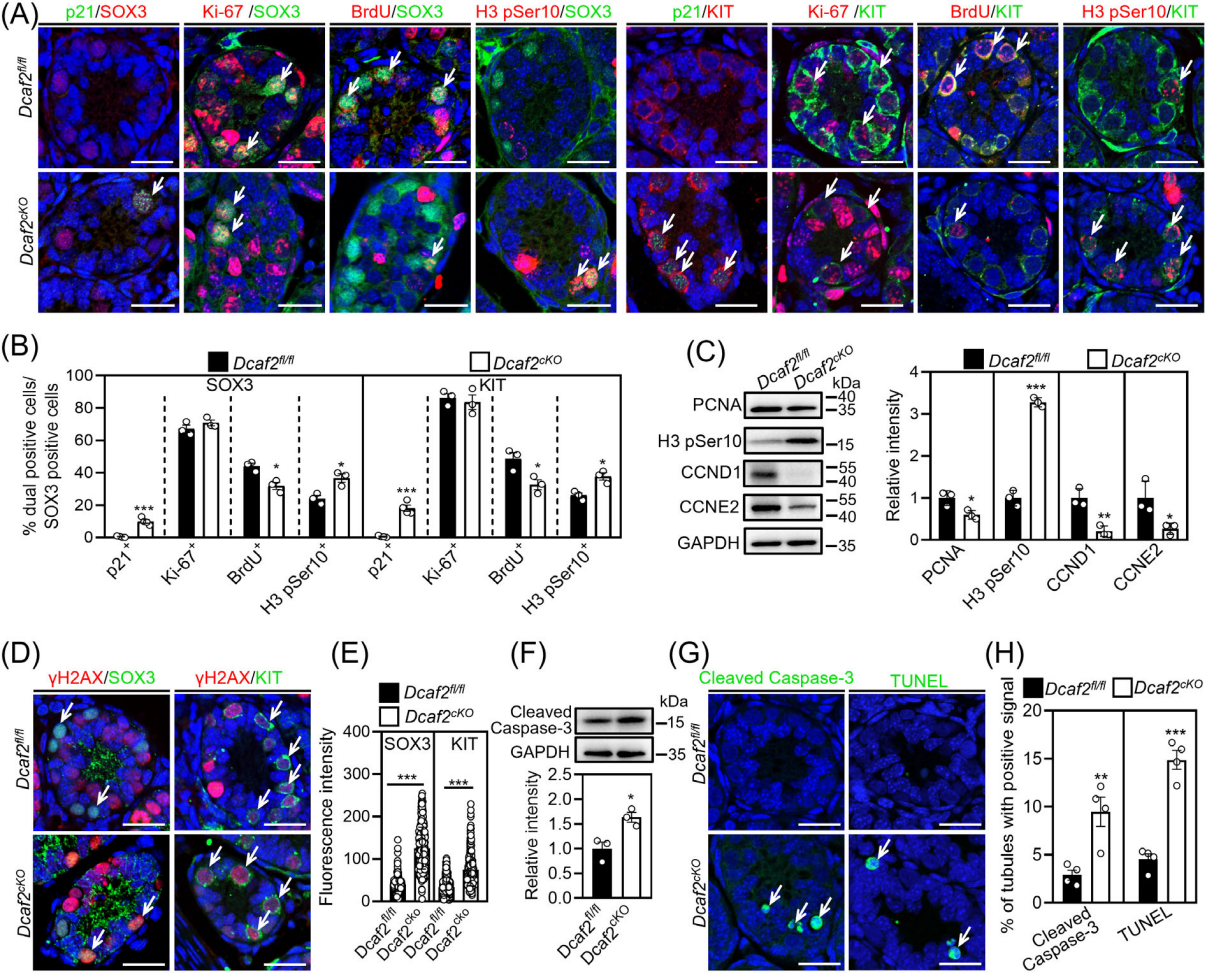
*Dcaf2* depletion attenuates proliferation and increases apoptosis of progenitor spermatogonia. (A) Double immunofluorescence staining of SOX3 or KIT with p21, Ki‐67, BrdU and H3 pSer10, respectively, in cKO and control testes at P9. Nuclei were counterstained with DAPI (blue). (B) Ratio of double immunofluorescence staining of SOX3^+^ and KIT^+^ cells in cKO and control testes at P9 (shown as %). (C) Western blot analysis of PCNA, H3 pSer10, CCNE2 and CCND1 levels in cKO and control testes. GAPDH was used as an internal control. (D) Double immunofluorescence staining of γH2AX with GFRα1 in cKO and control testes at P9. (E) Fluorescence intensity analysis of γH2AX in SOX3^+^ and KIT^+^ cells. (F) Western blotting analysis of cleaved caspase‐3 levels in cKO and control testes. (G) Immunofluorescence staining of cleaved caspase‐3 and TUNEL in cKO and control testes at P9. (H) Ratio of cleaved caspase‐3 and TUNEL positive signal tubule in cKO and control testes at P9 (shown as %). White arrowheads indicate representative double positive cells or apoptotic cells. For C and F, *n* = 3 independent experiments. For B and E, more than 300 SOX3^+^ or KIT^+^ cells from 3 to 4 independent experiments were scored in each group. For H, more than 440 tubules from 4 independent experiments were scored in each group. Scale bars; 25 μm. *p < 0.05, **p < 0.01, ***p < 0.001, ns; no significance.

We investigated the effect of *Dcaf2* depletion on spermatogonial proliferation using immunofluorescence staining and western blotting. In SOX3^+^ (progenitor spermatogonia) and KIT^+^ (differentiating spermatogonia) cells, the proportion of p21^+^ cells and H3 pSer10^+^ cells was significantly increased, the proportion of BrdU^+^ cells was significantly decreased, and the proportion of Ki‐67^+^ cells was not significantly different in cKO testes compared with controls (Figures 6A,B and S9A). In GFRα1^+^ cells (SSCs), the proportion of p21^+^ cells, H3 pSer10^+^ (G2/M phase marker) cells, BrdU^+^ cells and Ki‐67^+^ cells showed no difference between cKO and control testes (Figure S10A,B). The protein levels of PCNA, cyclin E2 (CCNE2) and cyclin D1 (CCND1) were significantly decreased, and the protein levels of H3 pSer10 were significantly increased in cKO testes compared with the controls (Figure 6C). These results suggest that *Dcaf2* depletion likely impairs proliferation and G2/M phase transition in progenitor spermatogonia. The impaired proliferation of progenitor spermatogonia may lead to defects in differentiation.^3^

In addition, the impact of *Dcaf2* depletion on DNA damage and apoptosis in spermatogonia was investigated using immunofluorescence staining and western blotting. The fluorescence intensity of γH2AX in SOX3^+^ and KIT^+^ cells, but not GFRα1^+^ cells, was significantly increased in cKO testes compared with the controls (Figures 6D,E and S11A–C). The protein levels of cleaved caspase‐3 and the percentage of tubules with cleaved caspase‐3‐ and TUNEL‐positive signals were significantly increased in cKO testes compared with the controls (Figure 6F–H and S11D). Thus, *Dcaf2* depletion increases DNA damage, which may further lead to the apoptosis of spermatogonia.

### 3.7 p21 and TDG overexpression increases DNA damage and cell apoptosis in GC‐1 cells

**FIGURE 7:**
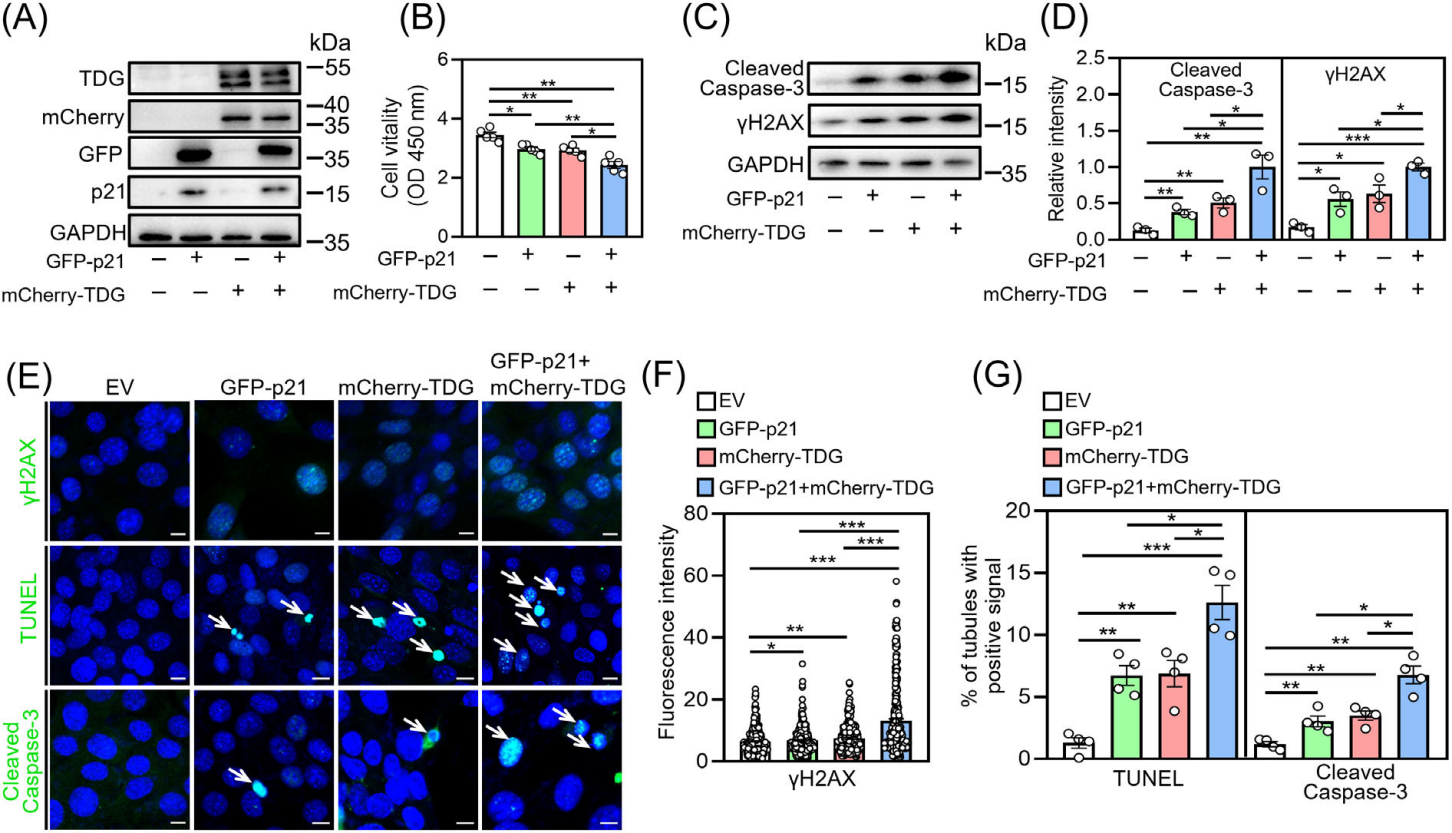
p21 and/or TDG overexpression attenuates proliferation and increases DNA damage and apoptosis in GC‐1 cells. (A) Western blot analysis for p21 and/or TDG expression in GC‐1 cells transfected with pcDNA3.1‐NC (EV), pcDNA3.1‐EGFP‐p21, pSC2‐mCherry‐TDG or pcDNA3.1‐EGFP‐p21 + pSC2‐mCherry‐TDG. (B) CCK‐8 assay for cell viability. (C, D) Western blot analysis of γH2AX and cleaved caspase‐3 expression in GC‐1 cells transfected with pcDNA3.1‐NC, pcDNA3.1‐EGFP‐p21, pSC2‐mcherry‐TDG or pcDNA3.1‐EGFP‐p21 + pSC2‐mCherry‐TDG. (E) TUNEL assay for apoptosis and immunofluorescence staining for γH2AX and cleaved caspase‐3. Nuclei were counterstained with DAPI (blue). White arrowheads indicate representative apoptotic cells. (F) Fluorescence intensity analysis of γH2AX in GC‐1 cells. (G) Ratio of cleaved caspase‐3 and TUNEL‐positive signals in GC‐1 cells. Scale bars; 10 μm. For B, *n* = 5 independent experiments. For D, *n* = 3 independent experiments. More than 300 cells (F) from 3 independent experiments and more than 800 cells (G) from 4 independent experiments were scored in each group. *p‐*values were determined by one‐way ANOVA followed by Tukey's test *p < 0.05, **p < 0.01, ***p < 0.001, ns; no significance.

To further explore whether the abnormal accumulation of p21 and TDG is associated with increased DNA damage and apoptosis in mouse spermatogonia, we transfected the mouse spermatogonial cell line GC‐1 with pcDNA3.1‐NC, pcDNA3.1‐EGFP‐p21, pSC2‐mCherry‐TDG and pcDNA3.1‐EGFP‐p21 plus pSC2‐mCherry‐TDG to overexpress p21 and/or TDG. Western blotting confirmed the overexpression of p21 and/or TDG (Figure 7A). Overexpression of p21 or TDG attenuated cell proliferation (Figure 7B) and increased DNA damage and apoptosis in GC‐1 cells (Figure 7C–G), which is exacerbated by co‐overexpression of p21 and TDG. These results indicate that the abnormal accumulation of p21 and TDG inhibits cell proliferation and promotes apoptosis in GC‐1 cells.

## 4 DISCUSSION

**FIGURE 8:**
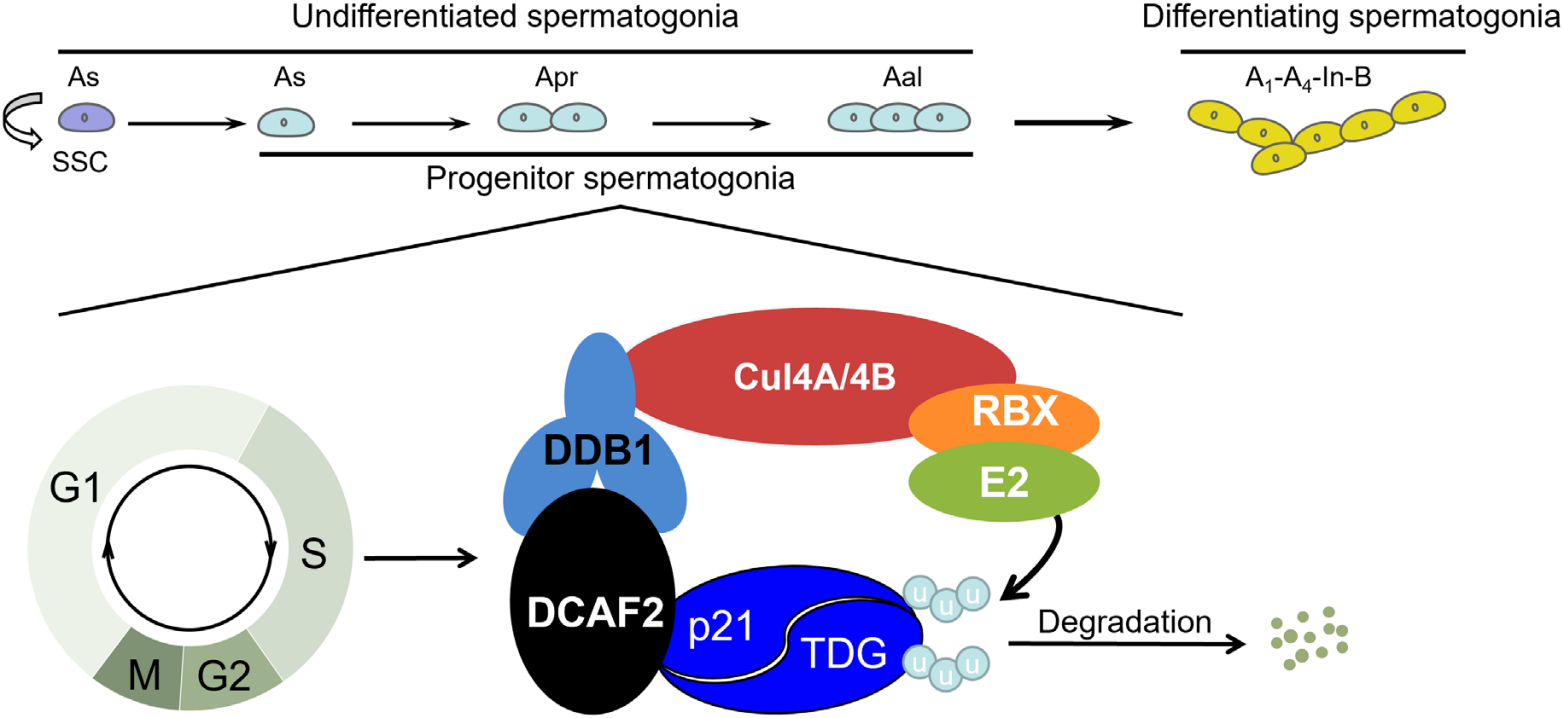
Schematic diagram of the CRL4^DCAF2^ ubiquitin complex in mouse progenitor spermatogonia. CUL4 serves as a scaffold that binds to the adaptor protein DDB1 and the substrate receptor protein DCAF2 at the N‐terminus and interacts with the ring‐finger protein RBX at the C‐terminus, thereby forming the CRL4^DCAF2^ E3 ligase complex. CRL4^DCAF2^ E3 ligase can promote ubiquitin transfer from RBX‐bound E2 ubiquitin‐conjugating enzymes to the DCAF2 substrate proteins p21 and TDG, promoting their degradation during the S phase. Degradation of these proteins maintains the proliferation and differentiation of progenitor spermatogonia. u, ubiquitin.

*Dcaf2* is highly expressed in germ cells of human and mouse testes. Depletion of *Dcaf2* in germ cells leads to abnormal accumulation of p21 and TDG in progenitor spermatogonia, leading to cell cycle arrest, DNA damage accumulation and apoptosis. These functional impairments lead to a reduction in progenitor spermatogonia and differentiating spermatogonia, leading to the failure of meiotic initiation and ultimately male infertility (Figure 8).

Depletion of *Dcaf2* in germ cells leads to a decrease in progenitor spermatogonia and differentiating spermatogonia, leading to failure of meiosis initiation and ultimately male infertility. The number of SSCs was unchanged in puberty cKO testes but increased in adult cKO testes, possibly due to the absence of differentiating spermatogonia. This is consistent with a previous study that the depletion of *Ck1α* in male germ cells sharply reduces progenitor spermatogonia and increases the number of SSCs on the seminiferous tubules as time progresses.^9^ Depletion of *Dcaf2* in germ cells did not affect the development of Sertoli cells and the integrity of the BTB, suggesting that the function of Sertoli cells was complete.^42^ This finding supports prior research indicating that the function of Sertoli cells remains unaffected even when all germ cells are eliminated through conditional knockout of *Nanos2*.^43^ The depletion of *Dcaf2* with *Stra8*‐Cre did not affect ovulation in female mice but caused infertility (data not shown), consistent with the results in which *Dcaf2* was depleted with *Gdf9*‐Cre.^33^ The female infertility caused by *Dcaf2* depletion in our study may be due to developmental arrest of the zygote.^33^ These results suggest that the depletion of *Dcaf2* does not affect meiosis in oocytes. DCAF2 is expressed in meiotic pro‐phase I spermatocytes. Further study is required to determine whether DCAF2 is involved in the spermatocyte meiosis.

The maintenance of cell cycle progression^44^ and genome stability^5^ is crucial for the proliferation and differentiation of spermatogonia. DCAF2 targets substrate proteins during the S phase to participate in the maintenance of cell cycle progression and genome stability.^45^ The depletion of *Dcaf2* leads to the abnormal accumulation of substrate proteins in B cells,^46^ intestinal epithelial cells,^47^ and zygotes^33^ during the S phase, ultimately leading to G2/M phase arrest and accumulation of DNA damage. In our study, *Dcaf2* depletion caused abnormal accumulation of p21 and TDG in progenitor spermatogonia, G2/M phase arrest, accumulation of DNA damage and apoptosis. Overexpression of p21 or TDG in the mouse spermatogonial cell line GC‐1 mimicked the phenotype of progenitor spermatogonia in *Dcaf2* cKO testes, which is exacerbated by co‐overexpression of p21 and TDG. Previous studies have shown that abnormal accumulation of p21 leads to G2/M phase arrest in renal tubular epithelial cells,^48^ kidney fibroblasts^49^ and zygotes.^50^ Abnormal TDG accumulation impairs cell proliferation and genomic stability in human embryonic kidney 293T cells^31^ and oocytes.^51^ Thus, we speculated that the abnormal accumulation of p21 due to *Dcaf2* depletion leads to cell cycle arrest in the G2/M phase, resulting in DNA damage and triggering apoptosis.^52^, ^53^ Abnormal accumulation of TDG is also involved in this process and may cause DNA damage through excessive DNA cleavage,^54^, ^55^ ultimately leading to apoptosis. Our results indicated that DCAF2 maintains appropriate p21 and TDG levels, which are critical for the proliferation and differentiation of progenitor spermatogonia. Notably, *Dcaf2* depletion led to a decrease in the proportion of progenitor spermatogonia (SOX3+) in the testes, which would mask DCAF2's substrate proteins with little difference in proteomics analysis. Although three substrate proteins (E2F1, CDT1 and KMT5A) with no significant differences in the proteomics were verified by western blotting, we cannot rule out that other substrate proteins are also involved in the defects of spermatogenesis in cKO mice. It would be a better way to use purified progenitor spermatogonia to perform proteomic screening in the future. Combined proteomic and transcriptomic analysis showed that a large number of genes were enriched in quadrants 6, 8 and 9, which may be due to a decrease in the regulation of protein ubiquitination caused by *Dcaf2* depletion. Genes enriched in 1, 2 and 4 may have been indirectly affected by *Dcaf2* depletion. In addition to, proteomic enrichment analysis showed that the downregulated proteins are related to cytoplasmic translation, which seems to be unrelated to the currently known DCAF2 ubiquitination substrate protein pathway. Thus, we speculate that the depletion of *Dcaf2* may lead to a decrease in cellular protein synthesis by non‐ubiquitination pathway.

The overexpression of DCAF2 has been reported in breast cancer,^56^ ovarian cancer,^57^ Alzheimer's disease^58^ and human papillomavirus.^59^ DCAF2 overexpression downregulates CDT1 in ovarian cancer,^57^ p21 in Alzheimer's disease^58^ and p21 and KMT5A in human papillomavirus^59^ to promote cell proliferation and survival. These studies indicate that DCAF2 targets different substrate proteins with tissue specificity, and maintains cell proliferation and survival through different mechanisms. In our study, DCAF2 targeted p21 and TDG to maintain normal cell cycle progression and genomic stability in mouse progenitor spermatogonia, ensuring their proliferation and differentiation. This study lays a foundation for the diagnosis and treatment of male infertility.

## AUTHOR CONTRIBUTIONS

Meijia Zhang and Hongwei Wei conceived the overall experimental plan. Heng‐Yu Fan and Zhijuan Wang participated in part of the experimental design. Longwei Gao and Yating Huang contributed to the genotype identification of mice and the isolation of mice testes. Yan‐Li Sun, Weiyong Wang and Shuang Liu provided advice on in vivo studies. Yashuang Weng and Huiyu Liu participated in the extraction and detection of mRNA and proteins. Meijia Zhang supervised the research and edited the article. All authors contributed feedback and approval to the final version of the article.

## CONFLICT OF INTEREST STATEMENT

The authors declare that they have no competing interests.

## Supporting information


**Data S1.** Supporting Information.

## Data Availability

The RNA‐seq data that support the findings of this study are openly available at the GEO repository (https://www.ncbi.nlm.nih.gov/geo/query/acc.cgi?acc=GSE259242). The mass spectrometry proteomics data have been deposited to the ProteomeXchange Consortium (https://proteomecentral.proteomexchange.org) via the iProX partner repository with the dataset identifier PXD050206.
