# Supplementary material for: DCAF2 regulates the proliferation and differentiation of mouse progenitor spermatogonia by targeting p21 and thymine DNA glycosylase
Source: Cell Prolif. 2024 Jun 4;57(10):e13676. doi: 10.1111/cpr.13676 (PMC11471390; doi:10.1111/cpr.13676)
Supplement: Supplementary file 1 — Data S1. Supporting Information. [file CPR-57-e13676-s001.doc]

**Supplemental Material**

Hongwei Wei, et al.


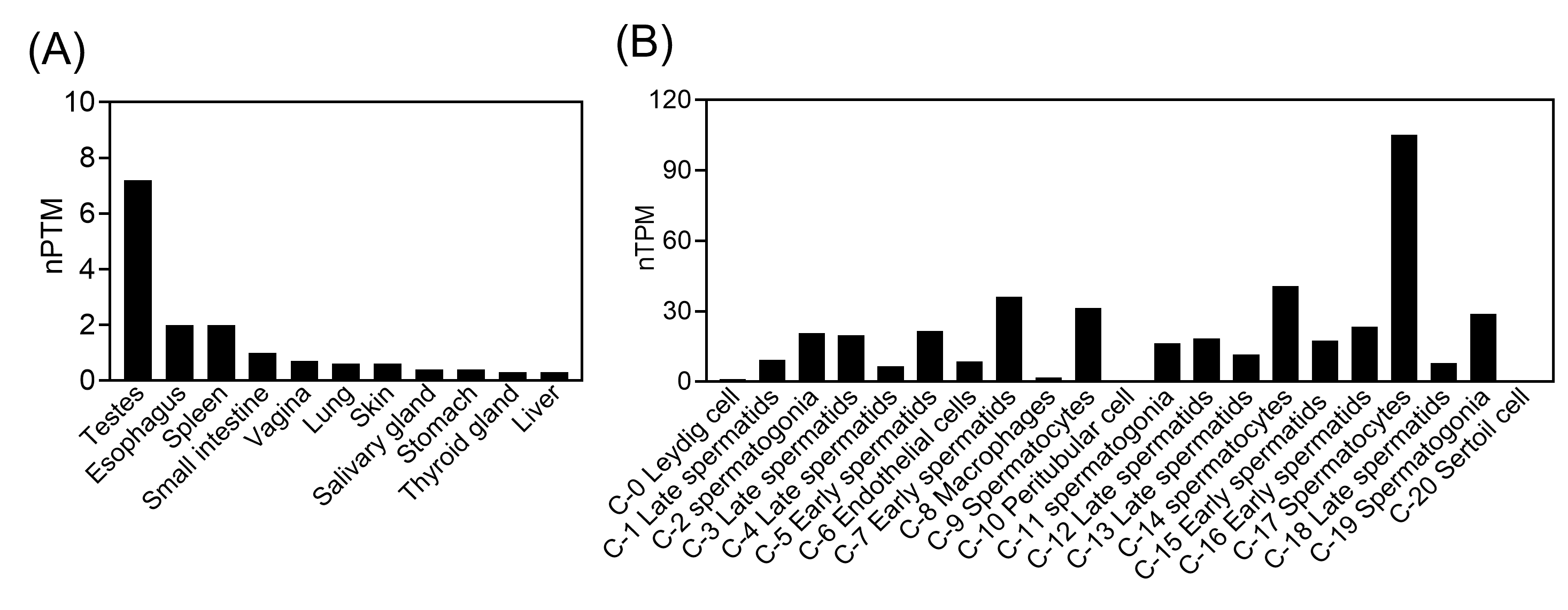
 **FIGURE S1 *Dcaf2* is highly expressed in germ cells of human testes.** (A, B) *Dcaf2* expression levels in different tissues (A) and in different testicular cells of human (B). Data from <https://www.proteinatlas.org/>.

**FIGURE S2 *Dcaf2* depletion in germ cells causes male sterility.** (A) Immunofluorescent staining for the Leydig cell marker HSD3β (green) in the Leydig cells isolated from P21 mice. Nuclei were counterstained by DAPI (blue). (B-D) Testes weight curve (B), morphological analysis (C), Hematoxylin and eosin (H&E) staining (D) of cKO and control testes in different stages. (E) Double-immunofluorescence staining of seminiferous tubule sections from adult mouse testes. DCAF2 (green), GFRα1 (a spermatogonial stem cells marker, red). Dotted circle indicate representative DCAF2^+^GFRα1^+^ Spermatogonial stem cells. Scale bars; A, 20 μm, C, 2000 μm, D, 50 μm, E, 100 μm. For B, n = 4 independent experiments. ***p* < 0.01, ****p* < 0.001, ns; no significance.


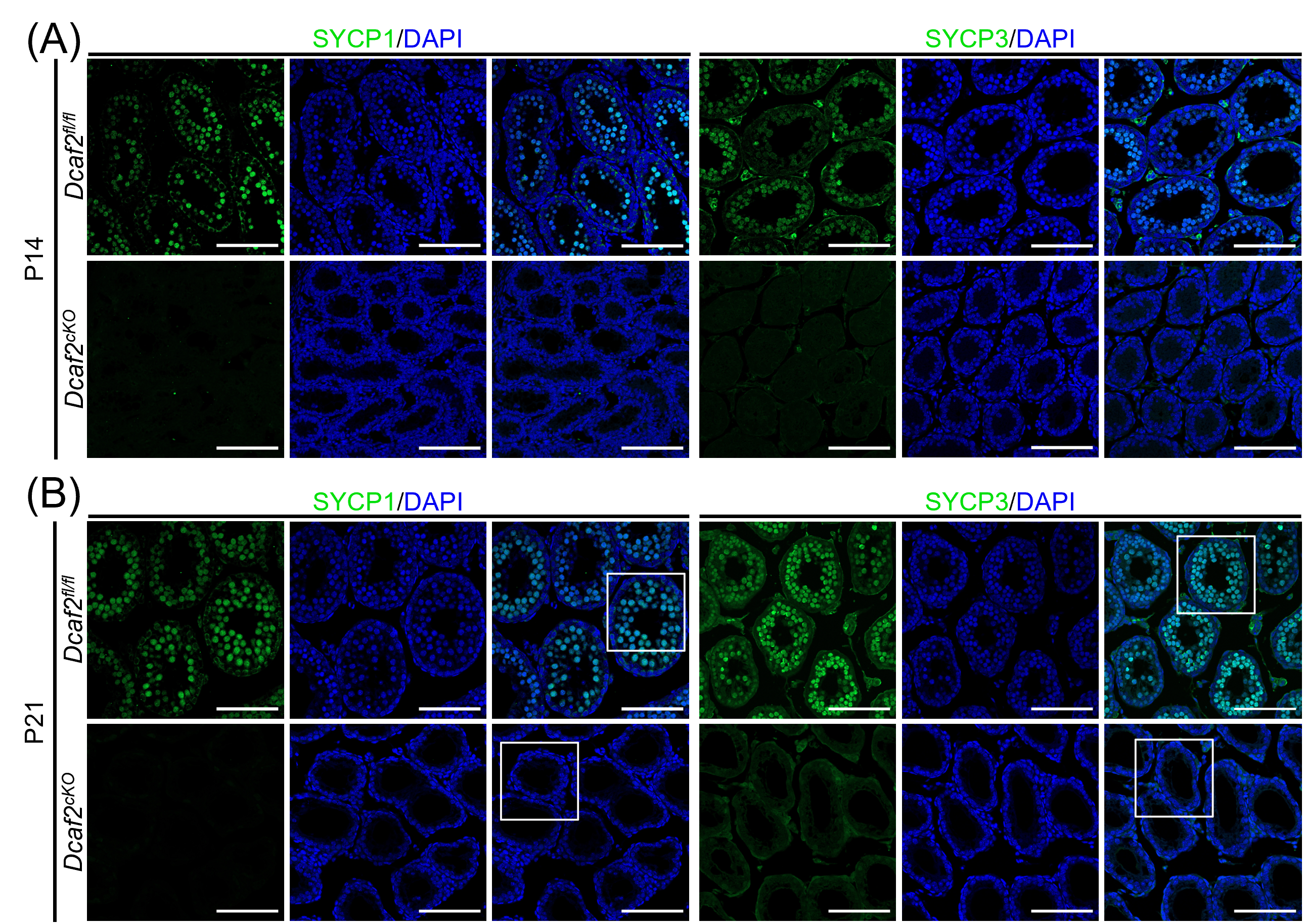


**FIGURE S3 Meiosis is completely blocked in cKO testes.** (A, B) Immunofluorescent staining of seminiferous tubule sections SYCP3 and SYCP1 in cKO and control testes at P14 (A) and P21 (B). Nuclei were counterstained with DAPI (blue). Scale bars; 100 μm.


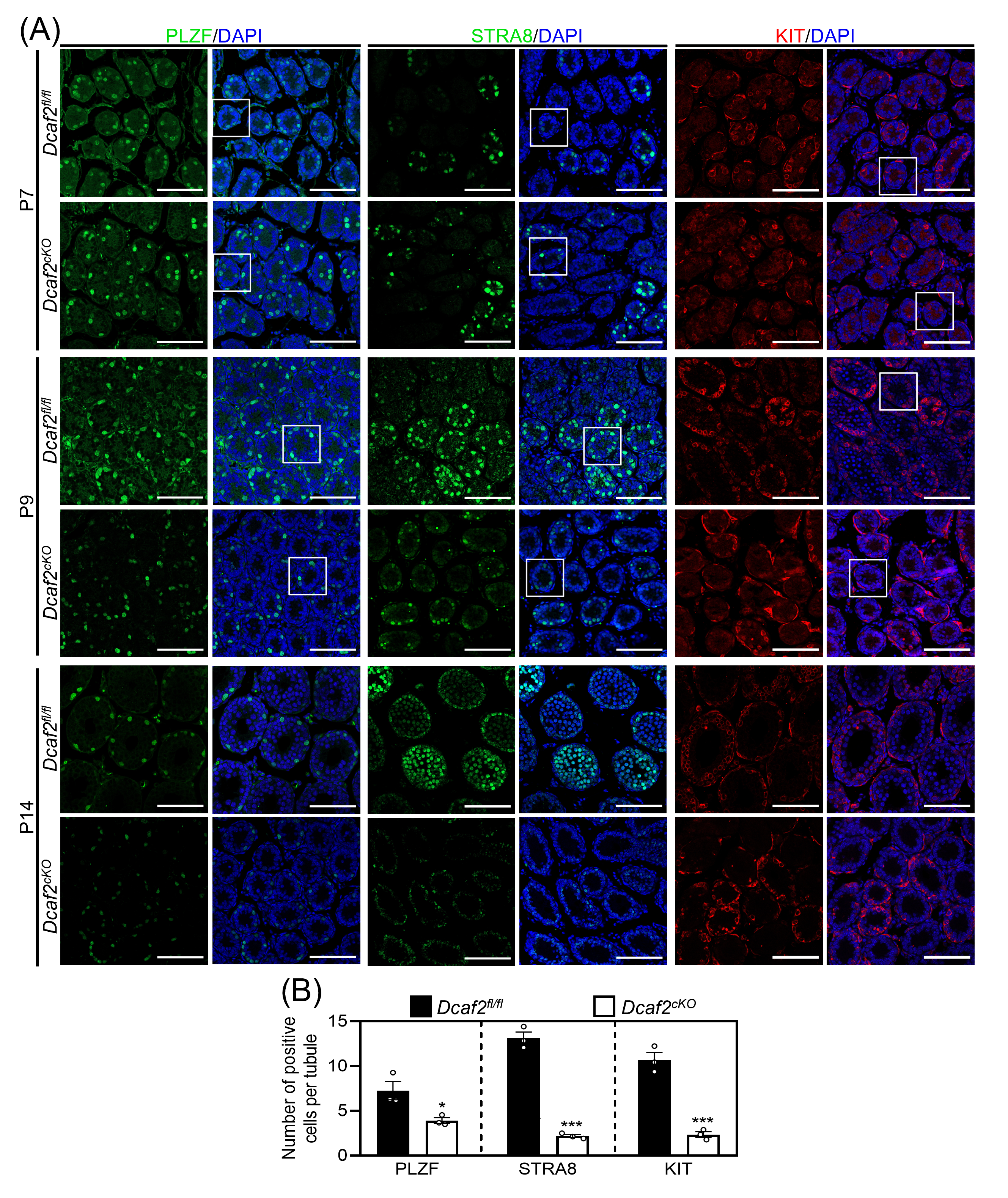
**FIGURE S4 PLZF^+^ undifferentiated spermatogonia and KIT^+^ differentiating spermatogonia are decreased in cKO testes.** (A) Immunofluorescent staining of PLZF, STRA8 and KIT in cKO and control testes in different stages. Nuclei were counterstained with DAPI (blue). Scale bars, 100 μm. (B) Number of PLZF^+^, STRA8^+^ and KIT^+^ cells per seminiferous tubule in cKO and control testes at P14. More than 120 tubules from 3 independent experiments were scored in each group. **p* < 0.05, ****p* < 0.001.


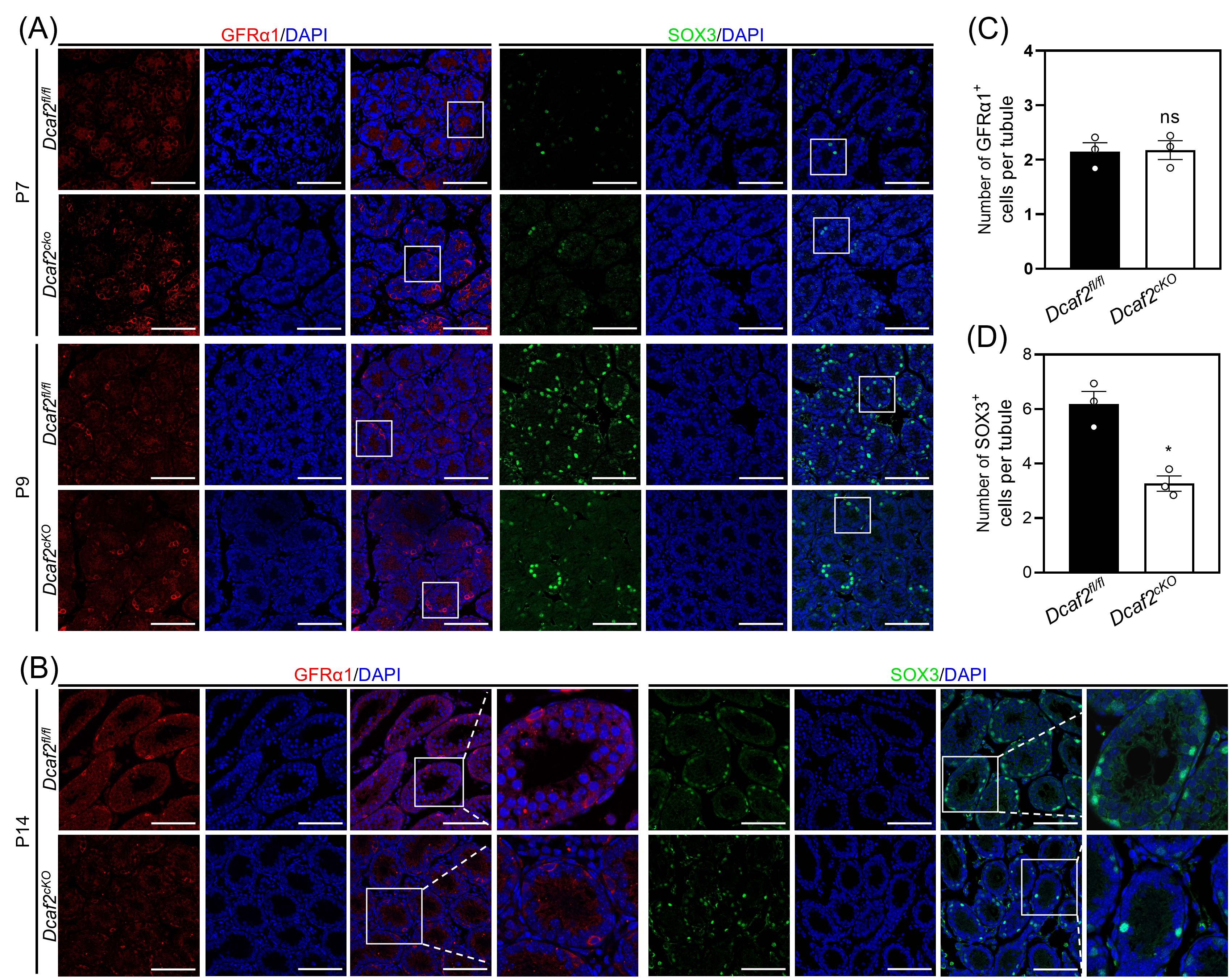
**FIGURE S5 SOX3^+^ progenitor spermatogonia are decreased in cKO testes.** (A, B) Immunofluorescent staining of GFRα1 and SOX3 in cKO and control testes in different stages. Nuclei were counterstained with DAPI (blue). Scale bars; 100 μm. (C, D) Numbers of GFRα1^+^ (C) and SOX3^+^ cells (D) per seminiferous tubule in cKO and control testes at P14. n = 3 independent experiments. More than 120 tubules from 3 independent experiments were scored in each group. ***p* < 0.01, ns; no significance.


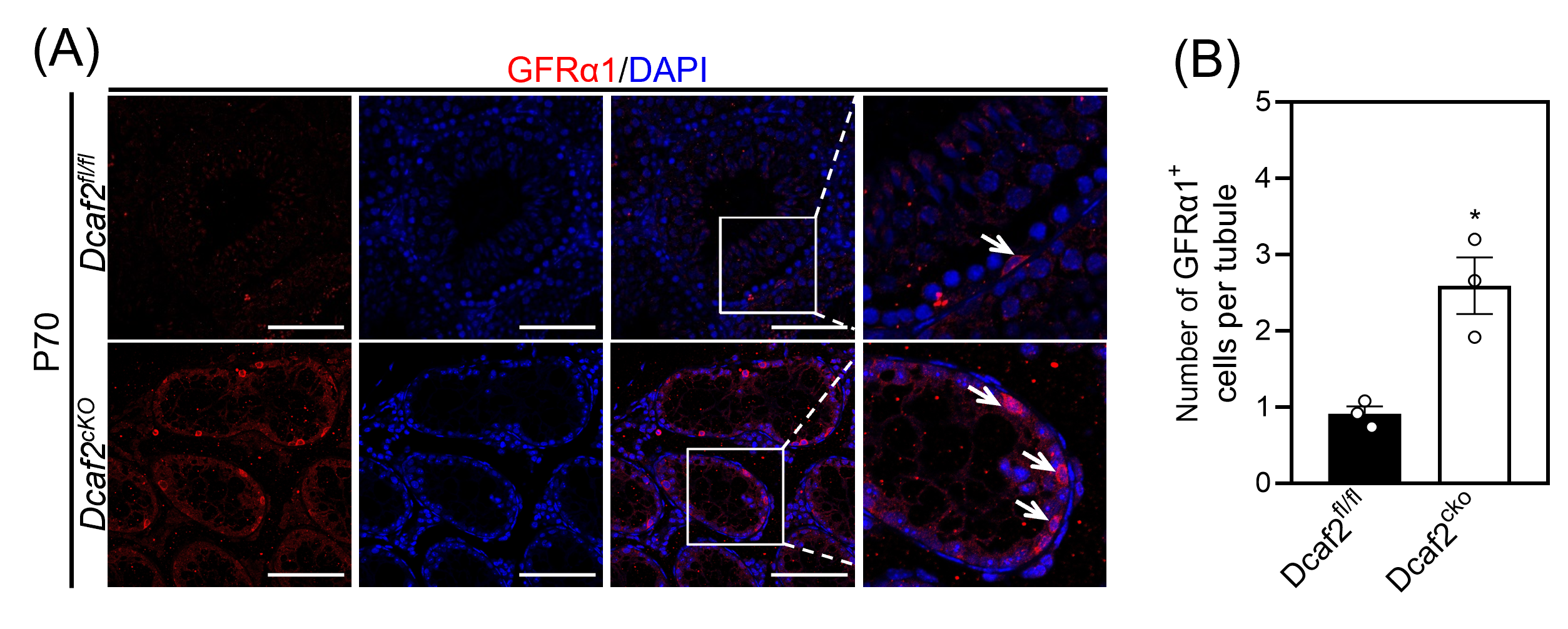


**FIGURE S6 GFRα1^+^ spermatogonial stem cells are increased in adult cKO testes.** (A) Immunofluorescence staining of GFRα1 in adult cKO and control testes. White arrowheads indicate representative GFRα1^+^ spermatogonial stem cells. Nuclei were counterstained with DAPI (blue). Scale bar; 100 μm. (B) Number of GFRα1^+^ cells per seminiferous tubule in adult cKO and control testes. More than 120 tubules from 3 independent experiments were scored in each group. **p* < 0.05.


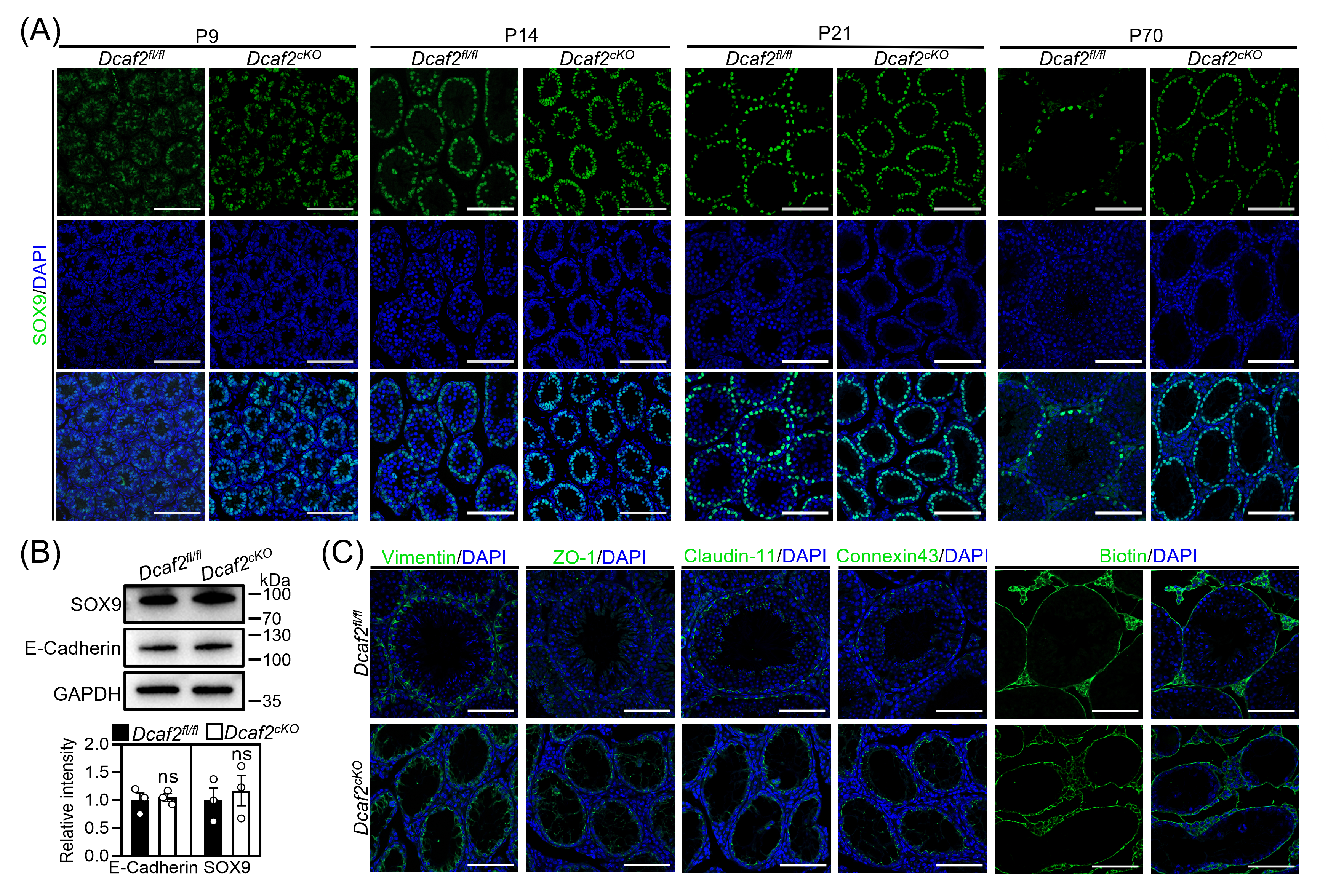
 **FIGURE S7 The number and function of SOX9^+^ Sertoli cells are not affected in adult cKO testes relative to controls.** (A) Immunofluorescent staining of SOX9 in cKO and control testes in different stages. Nuclei were counterstained with DAPI (blue). (B) Western blot analysis of E-Cadherin and SOX9 levels in adult cKO and control testes. GAPDH was used as an internal control. n = 3 independent experiments. (C) Immunofluorescent staining of vimentin, ZO-1, claudin11 and connexin43 and a biotin assay in adult cKO and control testes. Scale bars; 100 μm. ns; no significance.


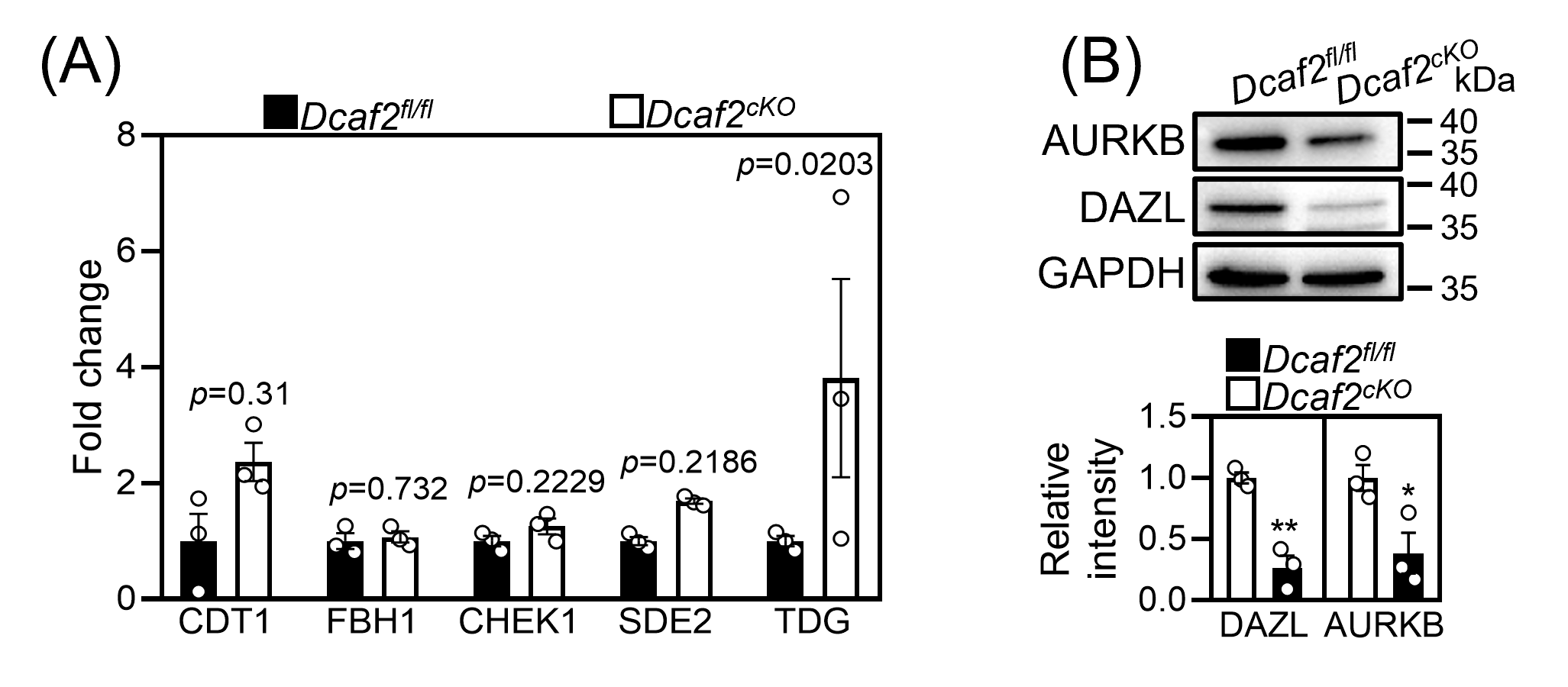


**FIGURE S8 Western blotting validates changes in the representative proteins selected from proteomic data.** (A) The protein levels of substrate proteins CDT1, FBH1, CHEK1, SDE2, and TDG from proteomics. (B) Western blot validating changes in the representative proteins selected from proteomic data. GAPDH was used as an internal control. n = 3 independent experiments. **p* < 0.05, ***p* < 0.01.


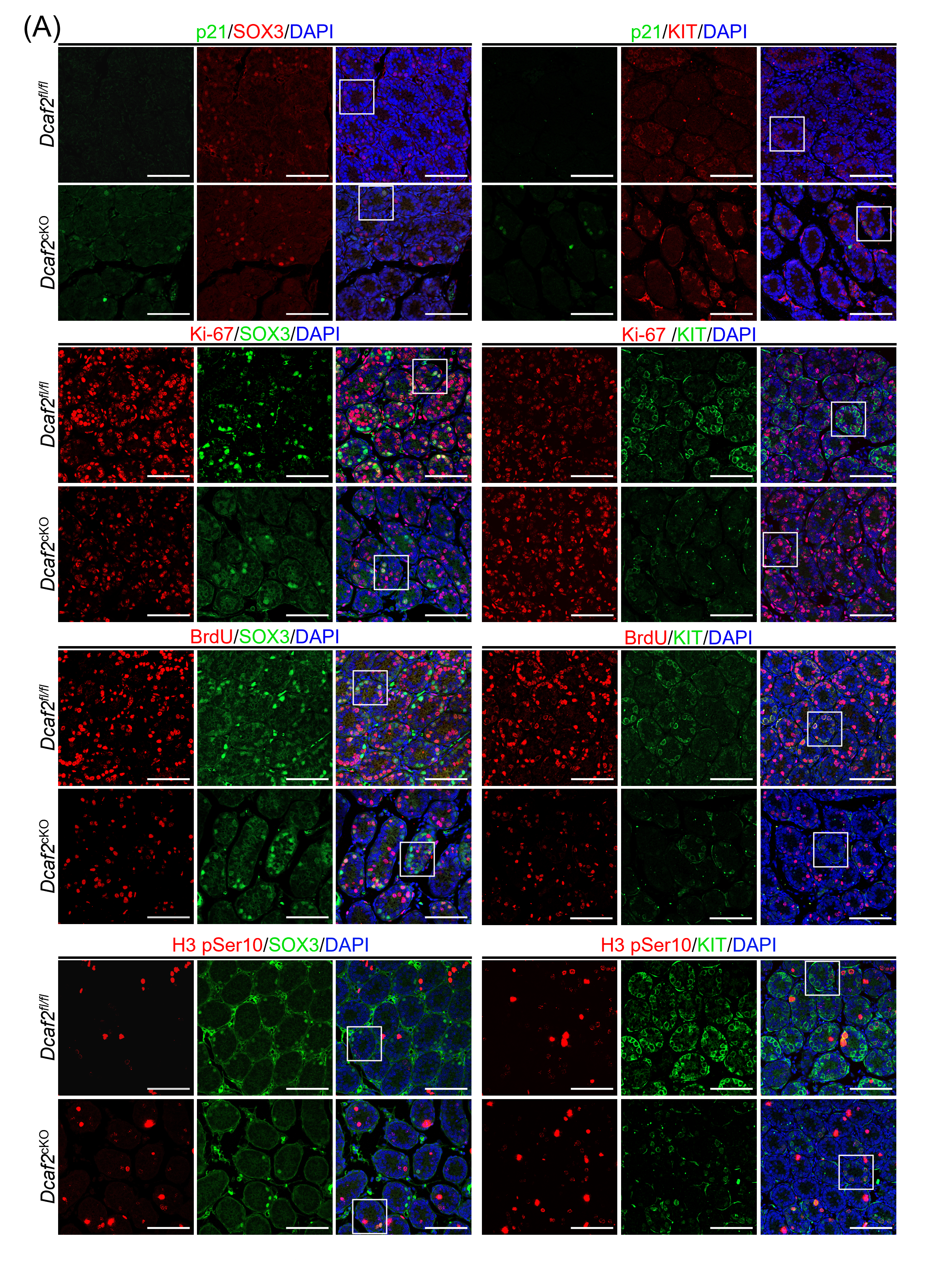
 **FIGURE S9 *Dcaf2* depletion attenuates proliferation of progenitor spermatogonia and differentiating spermatogonia.** (A) Double immunofluorescence staining of SOX3 or KIT with p21, Ki-67, BrdU and H3 pSer10, respectively, in cKO and control testes at P9. Nuclei were counterstained with DAPI (blue). Scale bars; 100 μm.


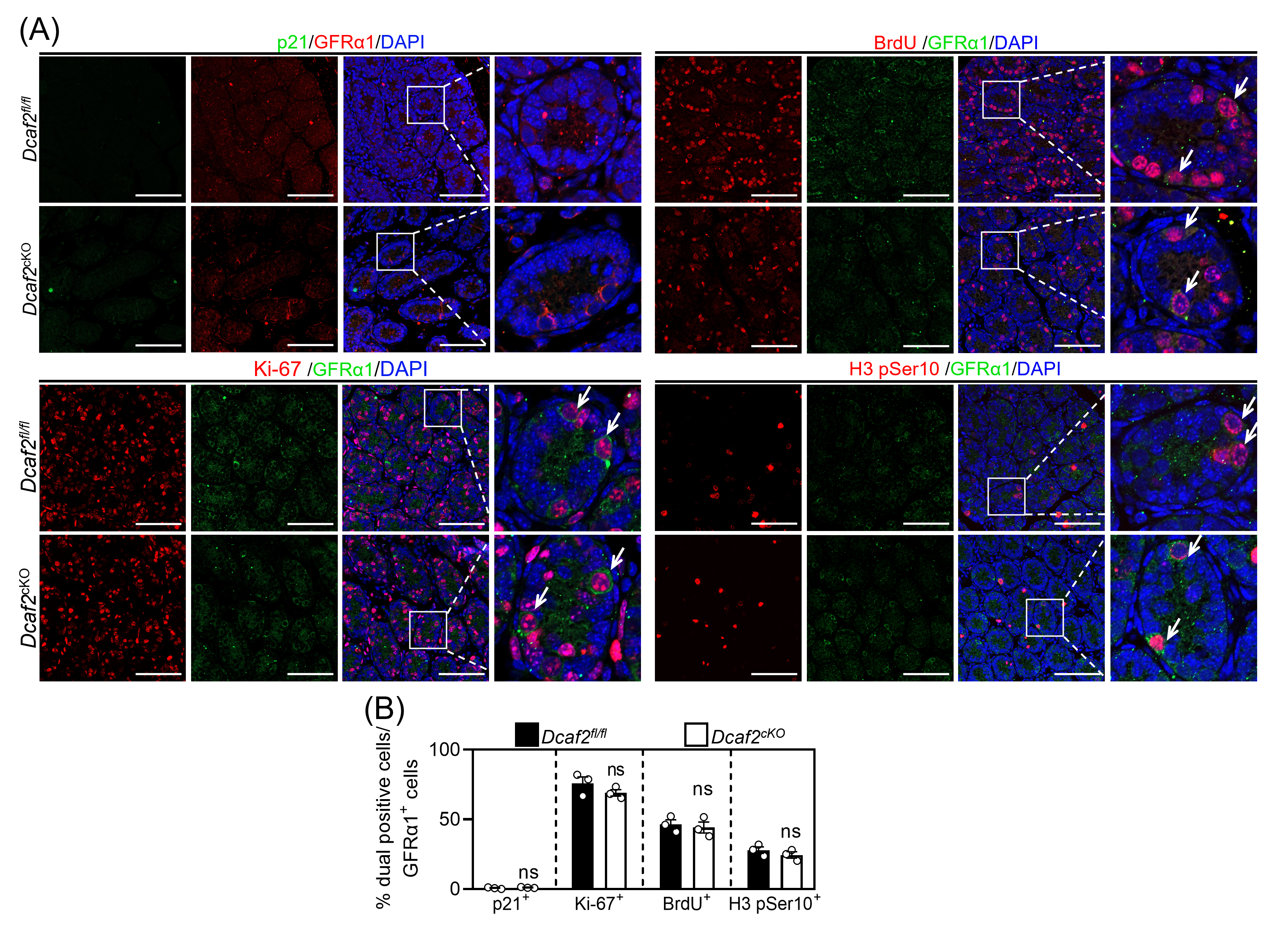
**FIGURE S10 *Dcaf2* depletion does not affect SSC proliferation.** (A) Immunofluorescent co-staining of GFRα1 with p21, Ki-67, BrdU and H3 pSer10, respectively, in cKO and control testes at P9. Nuclei were counterstained with DAPI (blue). White arrowheads indicate representative double-positive cells. Scale bar; 100 μm. (B) Quantification of the ratio of double-positive cells in GFRα1^+^ cells in cKO and controls testes at P9. More than 300 GFRα1^+^ cells from 3 independent experiments were scored in each group. ns, no significance.


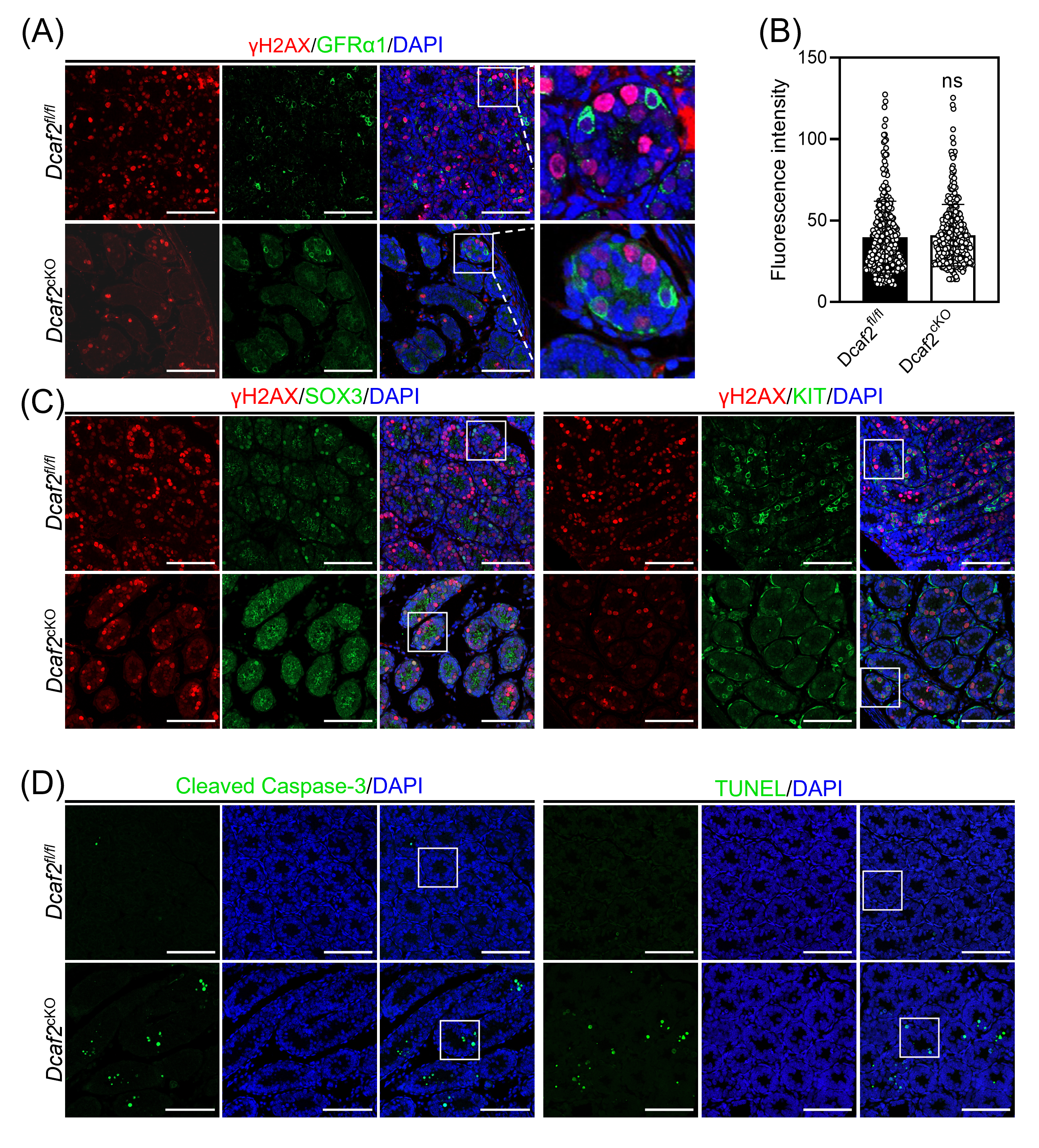


**FIGURE S11 *Dcaf2* depletion increases DNA damage and apoptosis of progenitor spermatogonia and differentiating spermatogonia.** (A) Double immunofluorescence staining of γH2AX with GFRα1 in cKO and control testes at P9. Nuclei were counterstained with DAPI (blue). (B) Fluorescence intensity analysis of γH2AX in GFRα1^+^ cells. A total of 300 cells from 3 independent experiments were scored in each group. (C) Double immunofluorescence staining of γH2AX with SOX3 and KIT, respectively, in cKO and control testes at P9. (D) Immunofluorescence staining of cleaved caspase-3 and TUNEL in cKO and control testes at P9. Scale bars, 100 μm. ns, no significance.

**T**able S1. Primers for PCR

| Primer name | Genes targeted | Application | Sequences (5’-3’) |
| --- | --- | --- | --- |
| FRT-F | *Dcaf2* | Identification of *Dcaf2* flox | 5’- CAGTGGCCTTGTGCATACTA-3’ |
| FRT-R |  |  | 5’-GTTACACCTGGTGTCCCAGATC-3’ |
| Cre-F | *Cre* | Identification of *Stra8-*Cre | 5’-ACTCCAAGCACTGGGCAGAA-3’ |
| Cre-R1 |  |  | 5’-GCCACCATAGCAGCATCAAA-3’ |
| Cre-R2 |  |  | 5’- CGTTTACGTCGCCGTCCAG-3’ |

**Table S2. Primers for qRT-PCR**

| Genes | Forwards (5’-3’) | Backwards (5’-3’) |
| --- | --- | --- |
| *Dcaf2* | GACATGCAAAGGCCACCAGT | GTCCCAGATCATAATGTTGCCG |
| *Tdg* | CAAGAGGACGCAAAGAAGATGG | TTGAAGCAATGCCACAAGGTT |
| *p21* | CCTGGTGATGTCCGACCTG | CCATGAGCGCATCGCAATC |
| *Kmt5a* | CAGACCAAACTGCACGACATC | CTTGCTTCGGTCCCCATAGT |
| *Cdt1* | AGAGCCCGAGTCCAAGAGAAT | CCTGATACTTGTAGGGTAGGACA |
| *Bcl6b* | GGCTACGTCCGAGAGTTCAC | CTTGTGCGCTCTTAGGGGT |
| *Cd82* | TGGACATCATTCGCAACTACAC | GCATGGGTAAGTGGTCTTGGTA |
| *Etv5* | CAAGTCCCTTTTATGGTCCCAG | ACTCTTCAGAATCGTGAGCCA |
| *Gfra1* | CACTCCTGGATTTGCTGATGT | AGTGTGCGGTACTTGGTGC |
| *Id4* | CAGTGCGATATGAACGACTGC | GACTTTCTTGTTGGGCGGGAT |
| *Lin28a* | GGCATCTGTAAGTGGTTCAACG | CCCTCCTTGAGGCTTCGGA |
| *Nanos3* | ATGGGGACTTTCAATCTTTGGAC | GTTTGCAGAATGAACATAAGCGT |
| *Neurog3* | CCAAGAGCGAGTTGGCACT | CGGGCCATAGAAGCTGTGG |
| *Sohlh1* | CGGGCCAATGAGGATTACAGA | TCCTGCGTTCTCTCTCGCT |
| *Sox3* | GAACGCATCAGGTGAGAGAAG | GTCGGAGTGGTGCTCAGG |
| *Sall4* | CCCTGGGAACTGCGATGAAG | TCAGAGAGACTAAAGAACTCGGC |
| *Zbtb16* | CTGCGGAAAACGGTTCCTG | GTGCCAGTATGGGTCTGTCT |
| *Dmrtb1* | GGAAGCAGTGCATCTGTGACA | GTGCTGATCCGGGTAGCAAA |
| *Dnmt3b* | AGCGGGTATGAGGAGTGCAT | GGGAGCATCCTTCGTGTCTG |
| *Kit* | GCCACGTCTCAGCCATCTG | GTCGGGATCAATGCACGTCA |
| *Prdm9* | CTGAATACAAGTGGCTCAGAACA | CCTCATAGGCAAGGCCCTTTC |
| *Stra8* | ACAACCTAAGGAAGGCAGTTTAC | GACCTCCTCTAAGCTGTTGGG |
| *Niban1* | AAGCAGACAACATTTGAAGCCC | ATCACCAGCTTACTCAGGACC |
| *Spta1* | ATGGGCTGTGATCGGAACTG | GTCTTCCCAATAAGCATGTCTCC |
| *Lats2* | GGACCCCAGGAATGAGCAG | CCCTCGTAGTTTGCACCACC |
| *Esr2* | TGTGCTATGGCCAACTTCTG | AGTAACAGGGCTGGCACAAC |
| *Pde11a* | AACAGGACCTACGATGAACAGG | TGAGGCAGATTCACCCTCGAT |
| *Rpl19* | CTGAAGGTCAAAGGGAATGTGTTC | TGGTCAGCCAGGAGCTTCTTG |

Table S3. List of primary antibodies used in immune detection

| Antibody | Catalog Code | Source | Host | Dilution | |
| --- | --- | --- | --- | --- | --- |
|  |  |  |  | IF | WB |
| AURKB | PTM-6125 | PTM Bio | Rabbit |  | 1:1000 |
| PLZF | sc-28319 | Santa Cruz Biotechnology | Mouse | 1:50 |  |
| SYCP3 | [sc-74569](https://www.scbt.com/zh/p/scp-3-antibody-d-1?requestFrom=search) | Santa Cruz Biotechnology | Mouse | 1:50 |  |
| p21 | ab188224 | Abcam | Rabbit | 1:50 | 1:1000  IP: 1:100 |
| CDT1 | [TD8171](http://www.ab-mart.com.cn/page.aspx?node= 65 &id= 23841) | Abmart | Rabbit |  | 1:1000 |
| TDG | NBP2-13423 | Novus Biologicals | Rabbit |  | 1:1000  IP: 1:100 |
| BrdU | ab1893 | Abcam | Sheep | 1:200 |  |
| BrdU | ab152095 | Abcam | Rabbit | 1:200 |  |
| Cleaved Caspase-3 | 9664 | Cell Signaling Technology | Rabbit | 1:50 | 1:1000 |
| STRA8 | ab49405 | Abcam | Rabbit | 1:200 | 1:1000 |
| DCAF2 | ab72264 | Abcam | Rabbit | 1:200 | 1:1000  IP: 1:100 |
| KMT5A | TA7553S | Abmart | Rabbit |  | 1:1000 |
| γH2AX | ab22551 | Abcam | Mouse | 1:200 | 1:1000 |
| SOX9 | ab185966 | Abcam | Rabbit | 1:200 | 1:1000 |
| Vimentin | 5741s | Cell Signaling Technology | Rabbit | 1:200 |  |
| ZO-1 | 40-2200 | Invitrogen | Rabbit | 1:200 |  |
| Connexin43 | 3512 | Cell Signaling Technology | Rabbit | 1:200 |  |
| E-Cadherin | 3195s | Cell Signaling Technology | Rabbit | 1:200 | 1:1000 |
| Claudin-11 | 36-4500 | Invitrogen | Rabbit | 1:200 |  |
| Ki-67 | 9129s | Cell Signaling Technology | Rabbit | 1:100 |  |
| PCNA | 2586 | Cell Signaling Technology | Mouse |  | 1:1000 |
| KIT | AF1356-SP | R&D Systems | Goat |  | 1:1000 |
| GFRα1 | ab233444 | Abcam | Rabbit | 1:100 | 1:1000 |
| GFRα1 | AF560-SP | R&D Systems | Goat | 1:100 |  |
| SOX3 | [sc-101155](https://www.scbt.com/zh/p/sox-3-antibody-16-c2?requestFrom=search) | Santa Cruz Biotechnology | Mouse | 1:50 |  |
| SOX3 | ab183606 | Abcam | Rabbit |  | 1：1000 |
| PNA | L21409 | Thermo Fisher Scientific |  | 1:200 |  |
| CCND1 | [sc-8396](https://www.scbt.com/zh/p/cyclin-d1-antibody-a-12?requestFrom=search) | Santa Cruz Biotechnology | Mouse |  | 1:500 |
| CCNE2 | sc-28351 | Santa Cruz Biotechnology | Mouse |  | 1:500 |
| DAZL | ab215718 | Abcam | Rabbit |  | 1:1000 |
| HSD3β | sc-515120 | Santa Cruz Biotechnology | Mouse | 1:50 |  |
| E2F1 | [sc-251](https://www.scbt.com/zh/p/e2f-1-antibody-kh95?requestFrom=search) | Santa Cruz Biotechnology | Mouse |  | 1:500 |
| TRA98 | ab82527 | Abcam | Rat | 1:200 |  |
| SYCP1 | ab175191 | Abcam | Rabbit | 1:200 |  |
| Ubi | PTM-5798 | PTM Bio | Mouse |  | 1:1000 |
| H3 pSer10 | ab267372 | Abcam | Rabbit | 1:200 | 1:1000 |
| GAPDH | 5174 | Cell Signaling Technology | Rabbit |  | 1:1000 |

IF: Immunofluorescence; WB: Western blotting; IP: Immunoprecipitation
